# Supplementary material for: Determinants of subjective total athletic ability
Source: PLoS One. 2025 May 28;20(5):e0324044. doi: 10.1371/journal.pone.0324044 (PMC12118886; doi:10.1371/journal.pone.0324044)
Supplement: S2 File — (DOCX) [file pone.0324044.s002.docx]

###############################################################################

#Initial Setting

###############################################################################

rm(list = ls())

setwd("/Users/sogakeishi/Desktop/2024_desktop/motor_skills/project_2/R")

set.seed(123)

###############################################################################

#Pre processing

###############################################################################

library(readxl)

library(readr)

library(tidyverse)

library(splines)

library(modelsummary)

library(ggplot2)

library(ggExtra)

library(psych)

library(corrplot)

library(dplyr)

library(psych)

library(car)

library(glmnet)

#library(plotmo)

library(ggExtra)

library(lmtest)

#Data loading

data <- read_excel("df_all_2.xlsx")

#Extracting column names

R.version

column_names <- colnames(data)

print(column_names)

#Consensus

data_con <- data %>%

filter(Consensus >= 1) %>%

filter(sex < 2)

nrow(data_con)

mean(data_con$age)

sd(data_con$age)

sum(data_con$sex)

#Data extraction

sex<-as.factor(data_con$sex)

living_highschool<-as.factor(data_con$living_highschool)

dominant_hand<-as.factor(data_con$dominant_hand)

dominant_foot<-as.factor(data_con$dominant_foot)

leisure_time_p1<-as.factor(data_con$leisure_time_p1)

leisure_time_p2<-as.factor(data_con$leisure_time_p2)

leisure_time_p3<-as.factor(data_con$leisure_time_p3)

sporthistory<-as.factor(data_con$sporthistory)

age <-data_con$age

birth_month_2 <-data_con$birth_month_2

father_income<-data_con$father_income

mother_income<-data_con$mother_income

walking_age<-data_con$walking_age

grit_total<-data_con$grit_total

res_total<-data_con$res_total

int_total<-data_con$int_total

sportkind_1yr<-data_con$sportkind_1yr

first_sportkind<-data_con$first_sportkind

competitive_results<-as.factor(data_con$competitive_results)

sub_abi<-data_con$sub_abi

fa_abi<-data_con$fa_abi

mo_abi<-data_con$mo_abi

volley<-data_con$volley

basket<-data_con$basket

badminton<-data_con$badmint

tennis<-data_con$tennis

baseball<-data_con$baseball

tabletennis<-data_con$tabletennis

soccer<-data_con$scccre

gymnastics<-data_con$gymna

short_distance<-data_con$short_field

long_distance<-data_con$long_field

total_ability<-data_con$total_ability

recognition_abi<-data_con$recognition_abi

compliments<-as.factor(data_con$compliments)

aqui_abi<-as.factor(data_con$aqui_abi)

bro_sis<-as.factor(data_con$bro_sis)

# Creating a dataframe

Data<- data.frame(

sex,

living_highschool,

dominant_hand,

dominant_foot,

leisure_time_p1,

leisure_time_p2,

leisure_time_p3,

age,

birth_month_2,

father_income,

mother_income,

walking_age,

grit_total,

res_total,

int_total,

sportkind_1yr,

first_sportkind,

competitive_results,

sub_abi,

fa_abi,

mo_abi,

volley,

basket,

badminton,

tennis,

baseball,

tabletennis,

soccer,

gymnastics,

short_distance,

long_distance,

total_ability,

recognition_abi,

compliments,

aqui_abi,

bro_sis,

sporthistory

)

nrow(Data)

#Checking for missing values

# Calculating the number of missing values in each column

na_counts_per_column <- colSums(is.na(Data))

print(na_counts_per_column)

###############################################################################

#Creating a correlation chart

###############################################################################

Data_3 <- data.frame(

sex,

volley,

basket,

badminton,

tennis,

baseball,

tabletennis,

soccer,

gymnastics,

short_distance,

long_distance,

sub_abi

)

Data_3$sex <- factor(Data_3$sex, levels = c(0, 1), labels = c("Women", "Men"))

library(GGally)

new_names <- sapply(names(Data_3), function(name) {

if(nchar(name) > 8) {

paste0(substr(name, 1, 5), "\n", substr(name, 6, nchar(name)))

} else {

name

}

})

names(Data_3) <- new_names

ggpairs(Data_3,

aes(colour = sex, alpha = 0.5),

upper = list(continuous = wrap("cor", size = 4)),

lower = list(continuous = "smooth", alpha = 0.3, size = 4, combo = "facetdensity"),

axisLabels = 'show') +

theme_bw(base_family = "Times New Roman") +

theme( axis.text.y = element_text(size = 18),

axis.text.x = element_text(size = 18),

strip.text = element_text(size = 15))

###############################################################################

#Linear model analysis and Making Figures

###############################################################################

#Model1

model1 <- lm(total_ability ~sex+age+birth_month_2+grit_total+res_total+int_total, data=Data)

summary(model1)

results_tab_1 <- msummary(model1,statistic = 'conf.int', conf_level = .95,stars=TRUE, fmt='%.3f',gof_omit='RMSE|AIC|BIC|Log.Lik.','data.frame')

#openxlsx::write.xlsx(results_tab_1, 'results_1.xlsx')

#vif(model1) #vif() allow to check a Multicollinearity

durbinWatsonTest(model1)

bptest(model1)

###############################################################################

#Cronbach's alpha coefficient

#grit

data_grit <- data_con %>%

select(grit_1,grit_2,grit_3,grit_4,grit_5,grit_6,grit_7,grit_8)

alpha(data_grit)

omega(data_grit,nfactors=2)

#resilience

data_res<- data_con %>%

select(res_1,res_2,res_3,res_4,res_5,res_6)

alpha(data_res)

omega(data_res,nfactors=1)

#Intelligence Scale

data_int<- data_con %>%

select(int_1,int_2,int_3,int_4,int_5,int_6,int_7,int_8,int_9,int_10)

alpha(data_int)

omega(data_int,nfactors=1)

###############################################################################

#Drawing Figures

library(tidyr)

library(dplyr)

library(forcats)

# Converting data to long format

Data_long_c <- pivot_longer(Data, cols = c(grit_total, res_total, int_total),

names_to = "Characteristics", values_to = "Value") %>%

mutate(Characteristics = fct_relevel(Characteristics, 'grit_total', 'res_total', 'int_total'))

colors_sex <- c("0" = "lightcoral", "1" = "lightskyblue")

scatter_plot_color <- ggplot(Data_long_c, aes(x = Value, y = total_ability, color = sex)) +

geom_point(size=3) +

scale_color_manual(values = colors_sex) +

geom_smooth(method = "lm", color = "black",

se = TRUE, size = 0.5, fill = "lightslategray", level = 0.95) +

facet_wrap(~ Characteristics, scales = "free_x",

labeller = as_labeller(c(grit_total = "Grit", res_total = "Resilience", int_total = "Intelligence"))) +

xlab(" ") +

ylab("Total Ability") +

theme_classic() +

theme_bw(base_family = "Times New Roman") +

theme(axis.title.x = element_text(size = 40),

axis.title.y = element_text(size = 40),

axis.text.x = element_text(size = 40, color = "black"),

axis.text.y = element_text(size = 40, color = "black"),

legend.position = "right",

panel.grid.major = element_blank(),

panel.grid.minor = element_blank(),

axis.line = element_line(size = 1, color = "black"),

strip.text = element_text(size = 40))

print(scatter_plot_color)

###############################################################################

#Model2

model2 <- lm(total_ability~sex+age+birth_month_2+leisure_time_p1+leisure_time_p2+leisure_time_p3, data=Data)

summary(model2)

results_tab_2 <- msummary(model2,statistic ='conf.int', conf_level = .95,stars=TRUE, fmt='%.3f',gof_omit='RMSE|AIC|BIC|Log.Lik.','data.frame')

#openxlsx::write.xlsx(results_tab_2, 'results_2.xlsx')

#vif(model2)

###############################################################################

#Drawing Figures

library(ggbeeswarm)

# Assuming 'Data' is your dataset

Data_long <- Data %>%

pivot_longer(

cols = c("leisure_time_p1", "leisure_time_p2", "leisure_time_p3"),

names_to = "leisure_time_category",

values_to = "leisure_time"

) %>%

mutate(leisure_time_category = case_when(

leisure_time_category == "leisure_time_p1" ~ "Lower grade",

leisure_time_category == "leisure_time_p2" ~ "Middle grade",

leisure_time_category == "leisure_time_p3" ~ "Higher grade",

TRUE ~ leisure_time_category

)) %>%

mutate(leisure_time_category = factor(leisure_time_category, levels = c("Lower grade", "Middle grade", "Higher grade"))) %>%

mutate(leisure_time = factor(case_when(

leisure_time == 1 ~ "Exercise",

leisure_time == 2 ~ "Game",

leisure_time == 3 ~ "Reading",

leisure_time == 4 ~ "Music",

leisure_time == 5 ~ "Study"

), levels = c("Exercise", "Game", "Reading", "Music", "Study")))

# Proceed with your plotting

ggplot(Data_long, aes(x=leisure_time, y=total_ability, color=leisure_time)) +

geom_boxplot(aes(fill=leisure_time), fill="white", size=0.5, color="black") +

geom_beeswarm(aes(group=leisure_time_category), alpha=0.7, size=3, cex = 1.5) +

scale_fill_viridis_d() +

facet_wrap(~leisure_time_category, scales = "free", ncol = 1) +

theme_classic(base_family = "Times New Roman") +

theme(

legend.position="none",

text = element_text(size=40),

axis.title = element_text(size=40, color="black"),

axis.text = element_text(size=40, color="black"),

panel.grid.major = element_blank(),

panel.grid.minor = element_blank(),

axis.line = element_line(size = 2, color="black"),

strip.text = element_text(size = 40, face = "bold"),

strip.background = element_blank()

) +

labs(x="Leisure Time", y="Total Ability")

###############################################################################

#Model3

model3 <- lm(total_ability~sex+age+birth_month_2+bro_sis, data=Data)

summary(model3)

results_tab_3 <- msummary(model3,statistic = 'conf.int', conf_level = .95,stars=TRUE, fmt='%.3f',gof_omit='RMSE|AIC|BIC|Log.Lik.','data.frame')

#openxlsx::write.xlsx(results_tab_3, 'results_3.xlsx')

#vif(model3)

###############################################################################

#Drawing Figures

Data_bro_sis <- Data %>%

mutate(bro_sis = factor(case_when(

bro_sis == 1 ~ "Only child",

bro_sis == 2 ~ "Oldest child",

bro_sis == 3 ~ "Middle child",

bro_sis == 4 ~ "Youngest child"

), levels = c("Only child", "Oldest child", "Middle child", "Youngest child")))

ggplot(Data_bro_sis, aes(x=bro_sis, y=total_ability, color=bro_sis)) +

geom_boxplot(aes(fill=bro_sis), fill="white", size=0.5, color="black")+

geom_beeswarm(aes(color=bro_sis), size=3, cex = 1.5) +

scale_fill_viridis_d() +

theme_classic(base_family = "Times New Roman") +

theme(

legend.position="none",

text = element_text(size=40),

axis.title = element_text(size=40, color="black"),

axis.text = element_text(size=40, color="black"),

panel.grid.major = element_blank(),

panel.grid.minor = element_blank(),

axis.line = element_line(size = 2, color="black"),

strip.text = element_text(size = 40, face = "bold"),

strip.background = element_blank()

) +

labs(x="Brother/Sister Status", y="Total Ability")

###############################################################################

#Model4

model4 <- lm(total_ability~sex+age+birth_month_2+sporthistory, data=Data)

summary(model4)

results_tab_4 <- msummary(model4,statistic = 'conf.int', conf_level = .95,stars=TRUE, fmt='%.3f',gof_omit='RMSE|AIC|BIC|Log.Lik.','data.frame')

#openxlsx::write.xlsx(results_tab_4, 'results_4.xlsx')

#vif(model4)

###############################################################################

#Drawing Figures

Data_sporthistory <- Data %>%

mutate(sporthistory = factor(case_when(

sporthistory == 0 ~ "No",

sporthistory == 1 ~ "Yes",

), levels = c("No", "Yes")))

ggplot(Data_sporthistory, aes(x=sporthistory, y=total_ability, color=sporthistory)) +

geom_boxplot(aes(fill=sporthistory), fill="white", size=0.5, color="black")+

geom_beeswarm(aes(color=sporthistory), size=3, cex = 1.5) +

scale_fill_viridis_d() +

theme_classic(base_family = "Times New Roman") +

theme(

legend.position="none",

text = element_text(size=40),

axis.title = element_text(size=40, color="black"),

axis.text = element_text(size=40, color="black"),

panel.grid.major = element_blank(),

panel.grid.minor = element_blank(),

axis.line = element_line(size = 2, color="black"),

strip.text = element_text(size = 40, face = "bold"),

strip.background = element_blank()

) +

labs(x="Exercise Experience", y="Total Ability")

###############################################################################

#Model5

Data_clean <- Data[!is.na(Data$sportkind_1yr), ]

nrow(Data_clean)

model5 <- lm(total_ability~sex+age+birth_month_2+sportkind_1yr+first_sportkind+competitive_results, data=Data_clean)

summary(model5)

results_tab_5 <- msummary(model5,statistic = 'conf.int', conf_level = .95,stars=TRUE, fmt='%.3f',gof_omit='RMSE|AIC|BIC|Log.Lik.','data.frame')

#openxlsx::write.xlsx(results_tab_5, 'results_5.xlsx')

#vif(model5)

results_tab_5

###############################################################################

#Drawing Figures

Data_long_sportkind <- pivot_longer(Data_clean, cols = c(sportkind_1yr, first_sportkind),

names_to = "Characteristics", values_to = "Value") %>%

mutate(Characteristics = fct_relevel(Characteristics, 'sportkind_1yr', 'first_sportkind')) # ここで順番を指定

colors_sex <- c("0" = "lightcoral", "1" = "lightskyblue")

scatter_plot_color_sportkind <- ggplot(Data_long_sportkind, aes(x = Value, y = total_ability, color = sex)) +

geom_point(size=3) +

scale_color_manual(values = colors_sex) +

geom_smooth(method = "lm", color = "black",

se = TRUE, size = 0.5, fill = "lightslategray", level = 0.95) +

facet_wrap(~ Characteristics, scales = "free_x",

labeller = as_labeller(c(sportkind_1yr = "Number of exercises \n learned over 1 year \n (Sport Kind)", first_sportkind = "Age at which they \n began learning to exercise \n (First Year) "))) +

xlab(" ") +

ylab("Total Ability") +

theme_classic() +

theme_bw(base_family = "Times New Roman") +

theme(axis.title.x = element_text(size = 40),

axis.title.y = element_text(size = 40),

axis.text.x = element_text(size = 40, color = "black"),

axis.text.y = element_text(size = 40, color = "black"),

legend.position = "right",

panel.grid.major = element_blank(),

panel.grid.minor = element_blank(),

axis.line = element_line(size = 1, color = "black"),

strip.text = element_text(size = 40))

print(scatter_plot_color_sportkind)

#competitive_results

Data_competitive_results <- Data_clean %>%

mutate(competitive_results = factor(case_when(

competitive_results == 1 ~ "Prefectural\nQualifier Level",

competitive_results == 2 ~ "Prefectural\nTournament Level",

competitive_results == 3 ~ "Prefectural\nAward Level",

competitive_results == 4 ~ "National\nTournament Level"

), levels = c("Prefectural\nQualifier Level", "Prefectural\nTournament Level",

"Prefectural\nAward Level", "National\nTournament Level")))

ggplot(Data_competitive_results, aes(x=competitive_results, y=total_ability, color=competitive_results)) +

geom_boxplot(aes(fill=competitive_results), fill="white", size=0.5, color="black") +

geom_beeswarm(aes(color=competitive_results), size=3, cex = 1.5) +

scale_fill_viridis_d() +

theme_classic(base_family = "Times New Roman") +

theme(

legend.position="none",

text = element_text(size=40),

axis.title = element_text(size=40, color="black"),

axis.text = element_text(size=40, color="black"),

panel.grid.major = element_blank(),

panel.grid.minor = element_blank(),

axis.line = element_line(size = 2, color="black"),

strip.text = element_text(size = 40, face = "bold"),

strip.background = element_blank()

) +

labs(x="Competitive Results", y="Total Ability")

###############################################################################

#Model6

Data_clean_2 <- Data[!(is.na(Data$father_income) | is.na(Data$mother_income)), ]

nrow(Data_clean_2)

#total_income

Data_clean_2$total_income <- Data_clean_2$father_income + Data_clean_2$mother_income

model6 <- lm(total_ability~sex+age+birth_month_2+total_income, data=Data_clean_2)

summary(model6)

results_tab_6 <- msummary(model6,statistic = 'conf.int', conf_level = .95,stars=TRUE, fmt='%.3f',gof_omit='RMSE|AIC|BIC|Log.Lik.','data.frame')

#openxlsx::write.xlsx(results_tab_6, 'results_6_total_income.xlsx')

#vif(model6)

results_tab_6

###############################################################################

#Drawing Figures

Data_long_income <- pivot_longer(Data_clean_2, cols = total_income,

names_to = "Characteristics", values_to = "Value")

colors_sex <- c("0" = "lightcoral", "1" = "lightskyblue")

scatter_plot_color_income <- ggplot(Data_long_income, aes(x = Value, y = total_ability, color = sex)) +

geom_point(size=3) +

scale_color_manual(values = colors_sex) +

geom_smooth(method = "lm", color = "black",

se = TRUE, size = 0.5, fill = "lightslategray", level = 0.95) +

facet_wrap(~ Characteristics, scales = "free_x",

labeller = as_labeller(c(total_income = "Income"))) +

xlab(" ") +

ylab("Total Ability") +

theme_classic() +

theme_bw(base_family = "Times New Roman") +

theme(axis.title.x = element_text(size = 40),

axis.title.y = element_text(size = 40),

axis.text.x = element_text(size = 40, color = "black"),

axis.text.y = element_text(size = 40, color = "black"),

legend.position = "right",

panel.grid.major = element_blank(),

panel.grid.minor = element_blank(),

axis.line = element_line(size = 1, color = "black"),

strip.text = element_text(size = 40))

print(scatter_plot_color_income)

###############################################################################

#Model7

Data_clean_4 <- Data[!(is.na(Data$fa_abi) | is.na(Data$mo_abi)), ]

nrow(Data_clean_4)

model7 <- lm(total_ability~sex+age+birth_month_2+fa_abi+mo_abi, data=Data_clean_4)

summary(model7)

results_tab_7 <- msummary(model7,statistic = 'conf.int', conf_level = .95,stars=TRUE, fmt='%.3f',gof_omit='RMSE|AIC|BIC|Log.Lik.','data.frame')

#openxlsx::write.xlsx(results_tab_7, 'results_7.xlsx')

#vif(model7)

results_tab_7

###############################################################################

#Drawing Figures

Data_long_fa_mo_abi <- pivot_longer(Data_clean_4, cols = c(fa_abi, mo_abi),

names_to = "Characteristics", values_to = "Value") %>%

mutate(Characteristics = fct_relevel(Characteristics, 'fa_abi', 'mo_abi')) # ここで順番を指定

colors_sex <- c("0" = "lightcoral", "1" = "lightskyblue")

scatter_plot_color_fa_mo_abi <- ggplot(Data_long_fa_mo_abi, aes(x = Value, y = total_ability, color = sex)) +

geom_point(size=3) +

scale_color_manual(values = colors_sex) +

geom_smooth(method = "lm", color = "black",

se = TRUE, size = 0.5, fill = "lightslategray", level = 0.95) +

facet_wrap(~ Characteristics, scales = "free_x",

labeller = as_labeller(c(fa_abi = "Father Ability", mo_abi = "Mother Ability"))) +

xlab(" ") +

ylab("Total Ability") +

theme_classic() +

theme_bw(base_family = "Times New Roman") +

theme(axis.title.x = element_text(size = 40),

axis.title.y = element_text(size = 40),

axis.text.x = element_text(size = 40, color = "black"),

axis.text.y = element_text(size = 40, color = "black"),

legend.position = "right",

panel.grid.major = element_blank(),

panel.grid.minor = element_blank(),

axis.line = element_line(size = 1, color = "black"),

strip.text = element_text(size = 40))

print(scatter_plot_color_fa_mo_abi)

###############################################################################

#Model8

model8 <- lm(total_ability~sex+age+birth_month_2+recognition_abi+compliments+aqui_abi, data=Data)

summary(model8)

results_tab_8 <- msummary(model8,statistic = 'conf.int', conf_level = .95,stars=TRUE, fmt='%.3f',gof_omit='RMSE|AIC|BIC|Log.Lik.','data.frame')

#openxlsx::write.xlsx(results_tab_8, 'results_8.xlsx')

#vif(model8)

results_tab_8

###############################################################################

#Drawing Figures

Data_long_rec <- pivot_longer(Data_clean_3, cols = recognition_abi,

names_to = "Characteristics", values_to = "Value")

colors_sex <- c("0" = "lightcoral", "1" = "lightskyblue")

scatter_plot_color_rec <- ggplot(Data_long_rec, aes(x = Value, y = total_ability, color = sex)) +

geom_point(size=3) +

scale_color_manual(values = colors_sex) +

geom_smooth(method = "lm", color = "black",

se = TRUE, size = 0.5, fill = "lightslategray", level = 0.95) +

facet_wrap(~ Characteristics, scales = "free_x",

labeller = as_labeller(c(recognition_abi = "Recognition Age"))) +

xlab(" ") +

ylab("Total Ability") +

theme_classic() +

theme_bw(base_family = "Times New Roman") +

theme(axis.title.x = element_text(size = 40),

axis.title.y = element_text(size = 40),

axis.text.x = element_text(size = 40, color = "black"),

axis.text.y = element_text(size = 40, color = "black"),

legend.position = "right",

panel.grid.major = element_blank(),

panel.grid.minor = element_blank(),

axis.line = element_line(size = 1, color = "black"),

strip.text = element_text(size = 40))

print(scatter_plot_color_rec)

#Compliments

Data_compliments <- Data %>%

mutate(compliments = factor(case_when(

compliments == 1 ~ "Never told",

compliments == 2 ~ "Not often said",

compliments == 3 ~ "Sometimes said",

compliments == 4 ~ "Often said",

), levels = c("Never told", "Not often said","Sometimes said","Often said")))

ggplot(Data_compliments, aes(x=compliments, y=total_ability, color=compliments)) +

geom_boxplot(aes(fill=compliments), fill="white", size=0.5, color="black")+

geom_beeswarm(aes(color=compliments), size=3, cex = 1.5) +

scale_fill_viridis_d() +

theme_classic(base_family = "Times New Roman") +

theme(

legend.position="none",

text = element_text(size=40),

axis.title = element_text(size=40, color="black"),

axis.text = element_text(size=40, color="black"),

panel.grid.major = element_blank(),

panel.grid.minor = element_blank(),

axis.line = element_line(size = 2, color="black"),

strip.text = element_text(size = 40, face = "bold"),

strip.background = element_blank()

) +

labs(x="Compliments", y="Total Ability")

#Motor Learning Speed

Data_aqui_abi <- Data %>%

mutate(aqui_abi = factor(case_when(

aqui_abi == 1 ~ "Too Slow",

aqui_abi == 2 ~ "Little Slow",

aqui_abi == 3 ~ "Average",

aqui_abi == 4 ~ "Little Fast",

aqui_abi == 5 ~ "Too Fast"

), levels = c("Too Slow", "Little Slow","Average","Little Fast", "Too Fast")))

ggplot(Data_aqui_abi, aes(x=aqui_abi, y=total_ability, color=aqui_abi)) +

geom_boxplot(aes(fill=aqui_abi), fill="white", size=0.5, color="black")+

geom_beeswarm(aes(color=aqui_abi), size=3, cex = 1.5) +

scale_fill_viridis_d() +

theme_classic(base_family = "Times New Roman") +

theme(

legend.position="none",

text = element_text(size=40),

axis.title = element_text(size=40, color="black"),

axis.text = element_text(size=40, color="black"),

panel.grid.major = element_blank(),

panel.grid.minor = element_blank(),

axis.line = element_line(size = 2, color="black"),

strip.text = element_text(size = 40, face = "bold"),

strip.background = element_blank()

) +

labs(x="Motore Learning Speed", y="Total Ability")

###############################################################################

#skewness&kurtosis

###############################################################################

library(moments)

# Create a function to generate histograms for each variable

create_hist_with_stats <- function(data, variable_name) {

# Calculation of skewness and kurtosis

sk <- round(skewness(data), 2)

ku <- round(kurtosis(data), 2)

# Creating histograms

p <- ggplot(data.frame(x = data), aes(x = x)) +

geom_histogram(aes(y = ..density..), bins = 30, fill = "lightblue", color = "black") +

geom_density(color = "red", linewidth = 1) +

annotate("label",

x = max(data),

y = max(density(data)$y),

label = sprintf("Skewness: %0.2f\nKurtosis: %0.2f", sk, ku),

hjust = 0.75,

vjust = -1.95,

size = 6,

label.padding = unit(0.5, "lines"),

label.size = 0.5,

label.r = unit(0.15, "lines"),

fill = "white") +

labs(title = variable_name,

x = "",

y = "Density")+

theme_bw(base_family = "Times New Roman") +

theme( axis.text.y = element_text(size = 25),

axis.text.x = element_text(size = 25),

strip.text = element_text(size = 25),

axis.title.x = element_text(size = 25),

axis.title.y = element_text(size = 25),

panel.background = element_rect(fill = "white"),

plot.background = element_rect(fill = "white"),

panel.grid.major = element_blank(),

panel.grid.minor = element_blank()

)

return(p)

}

create_hist_with_stats(Data$recognition_abi,"recognition_abi")

#############################################################################

#durbinWatsonTest&bptest

#############################################################################

durbinWatsonTest(model9)

bptest(model9)

#############################################################################

#categorical model

#############################################################################

options(contrasts = c("contr.sum", "contr.sum"))

Anova_model <- Anova(model2, type = "III")

Anova_model

library(kableExtra)

# Function to adjust the display of p-values

format_pvalue <- function(p) {

case_when(

p < 0.001 ~ "< 0.001***",

p < 0.01 ~ sprintf("%.3f**", p),

p < 0.05 ~ sprintf("%.3f*", p),

TRUE ~ sprintf("%.3f", p)

)

}

# Processing a DataFrame

anova_table <- as.data.frame(Anova_model) %>%

rownames_to_column(var = "Variable") %>%

mutate(`Pr(>F)` = sapply(`Pr(>F)`, format_pvalue)) # p値を整形

# Creating a table

kable(anova_table,

digits = c(0, 2, 0, 2, 3),

col.names = c("Variable", "Sum of Squares", "df", "F Value", "p-value"),

align = c("l", "r", "r", "r", "r")) %>%

kable_styling(bootstrap_options = c("striped", "hover"),

full_width = FALSE) %>%

row_spec(0, bold = TRUE) %>%

footnote(

symbol = c("*p < 0.05", "**p < 0.01", "***p < 0.001")

)

summary(model2)

#############################################################################

# Calculation of effect size

#############################################################################

# Calculate and output the effect size for each model

for (i in 1:8) {

# Generate model names

model_name <- paste0("model", i)

# Retrieve the model

model <- get(model_name)

# Retrieve R^2

summary_model <- summary(model)

R2 <- summary_model$r.squared

# Calculation of Cohen's f^2

f2 <- R2 / (1 - R2)

# Resuls

cat("Model", i, "Cohen's f^2:", f2, "\n")

}
