# Supplementary material for: Determinants of subjective total athletic ability
Source: PLoS One. 2025 May 28;20(5):e0324044. doi: 10.1371/journal.pone.0324044 (PMC12118886; doi:10.1371/journal.pone.0324044)
Supplement: S3 File — (PPTX) [file pone.0324044.s003.pptx]

## Slide 1
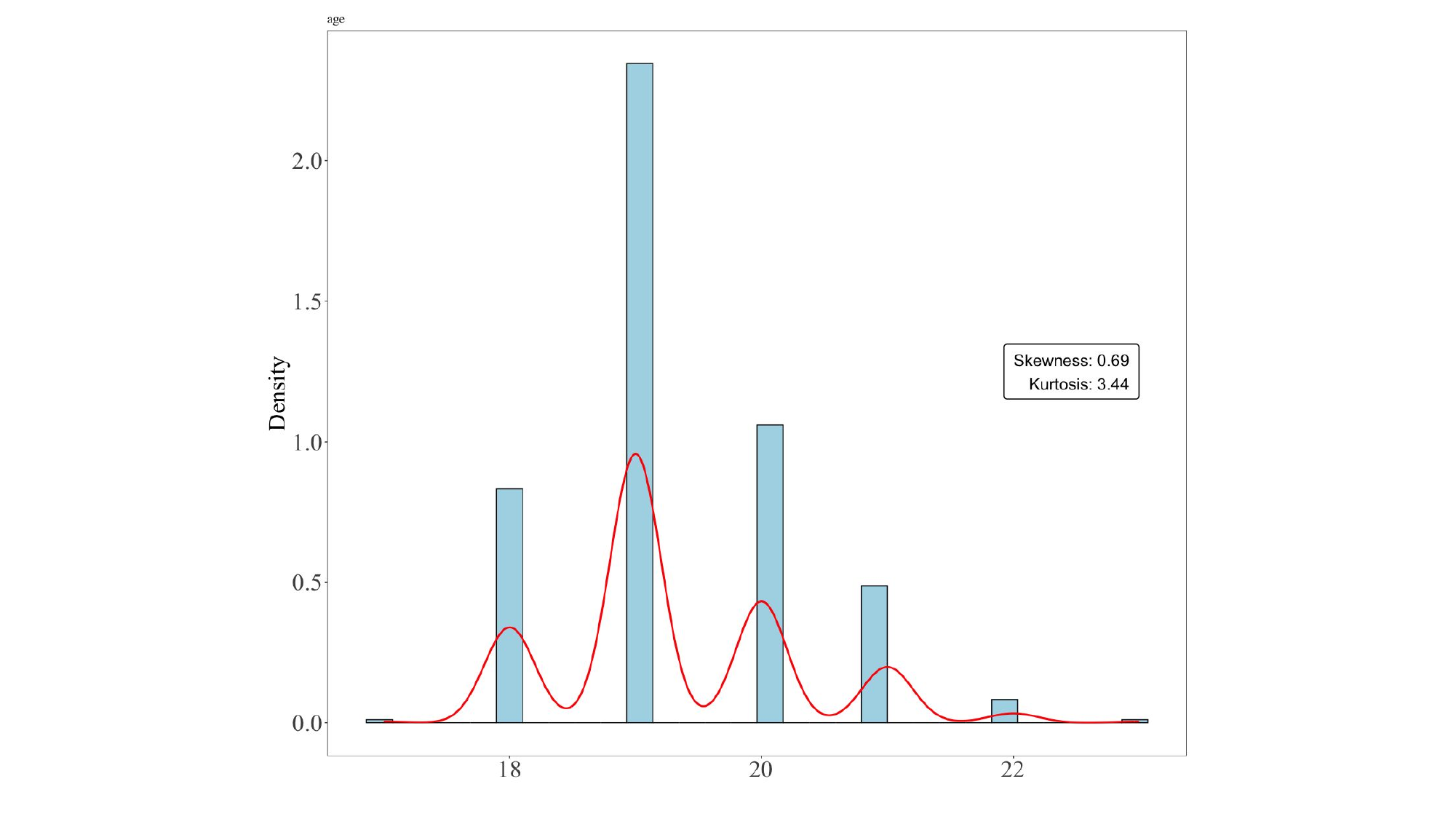

## Slide 2
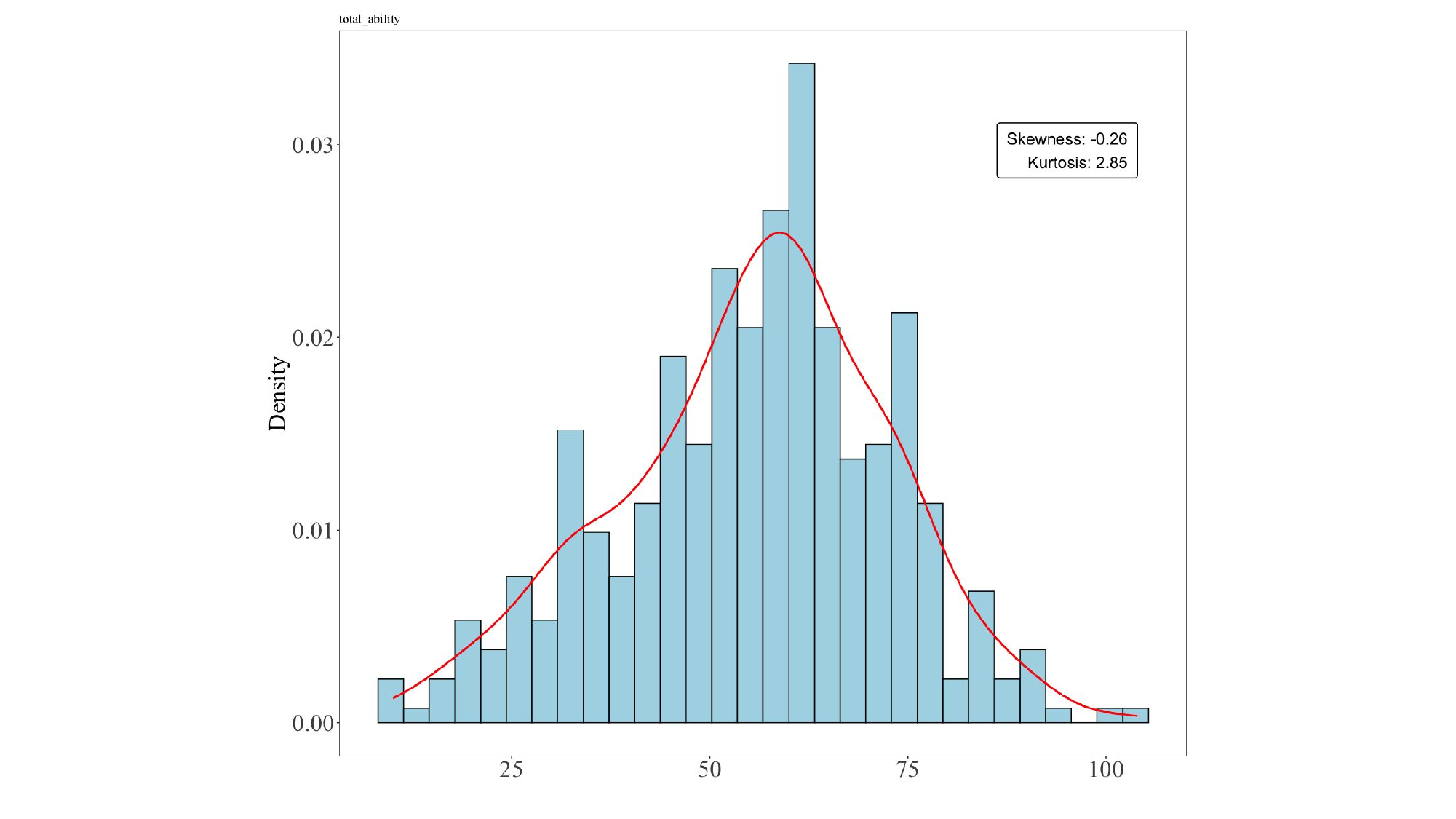

## Slide 3
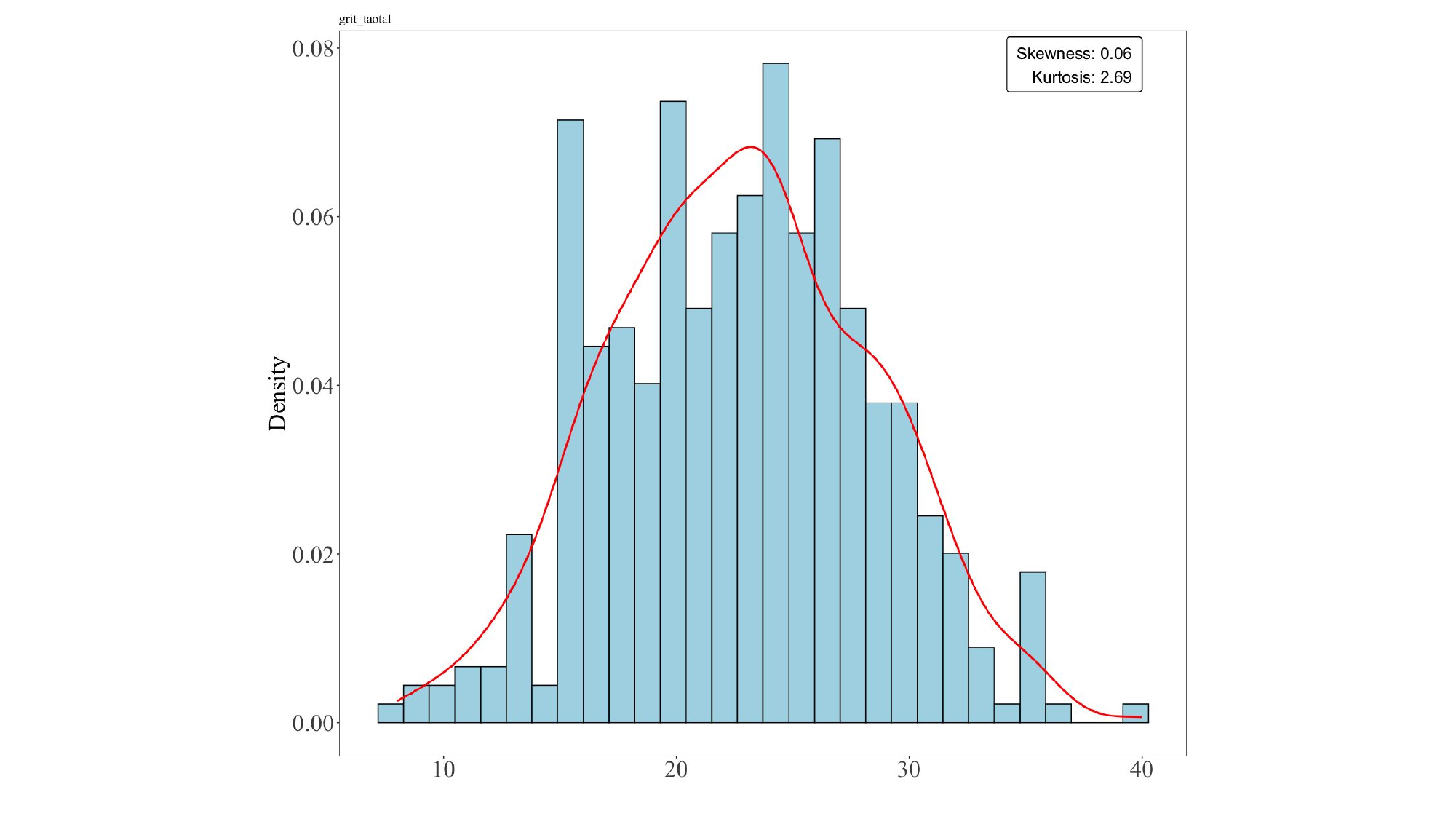

## Slide 4
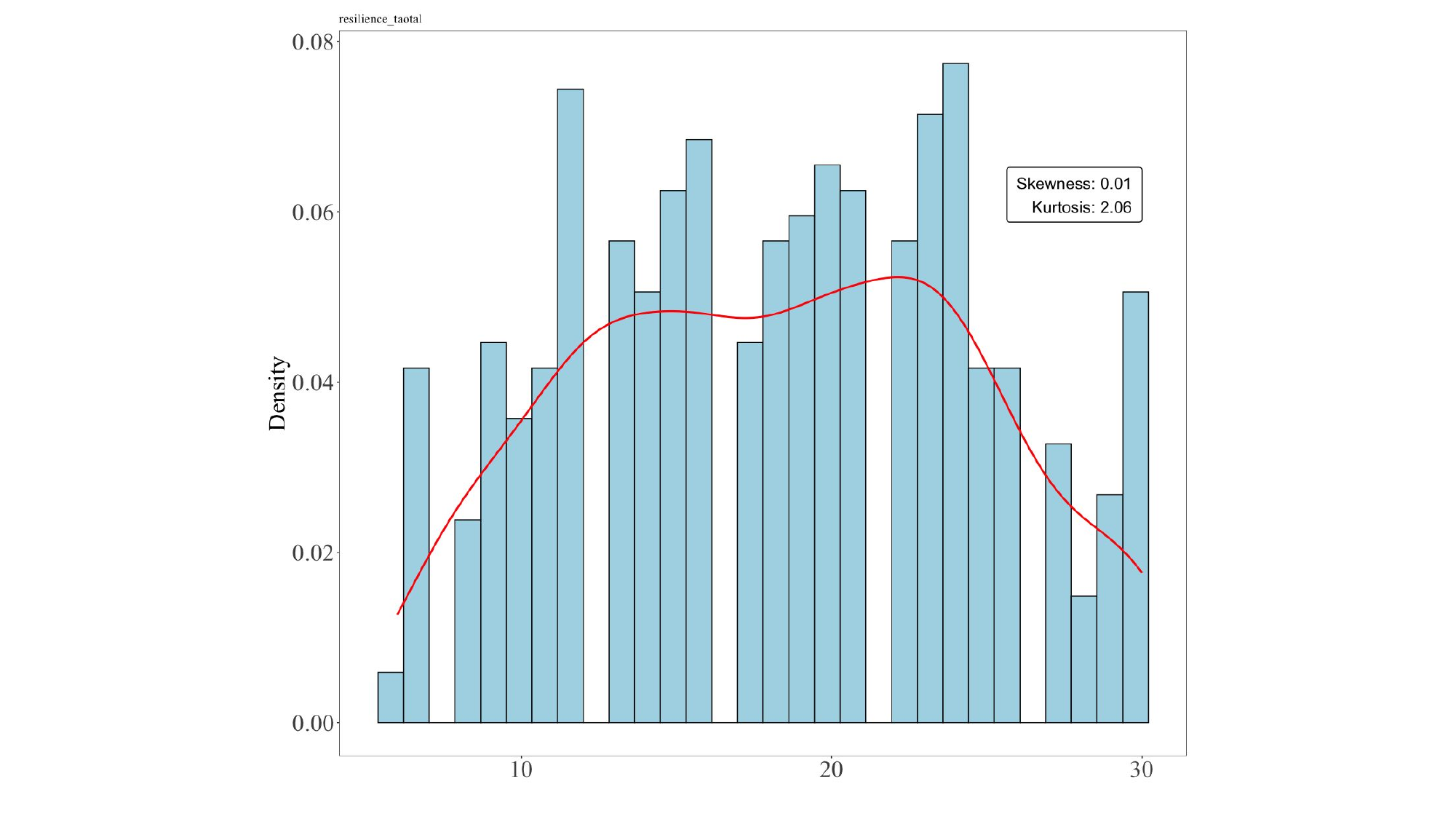

## Slide 5
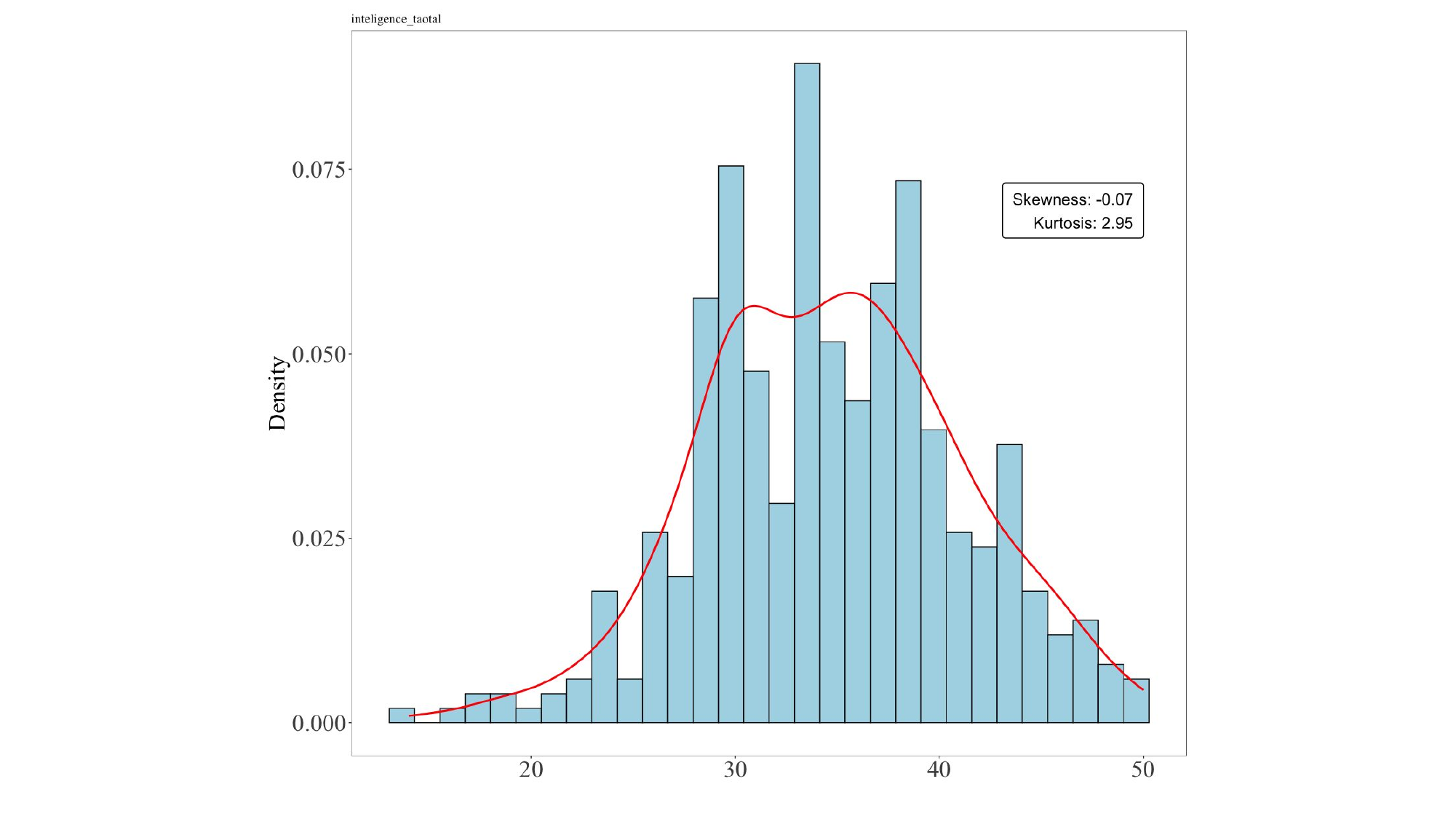

## Slide 6
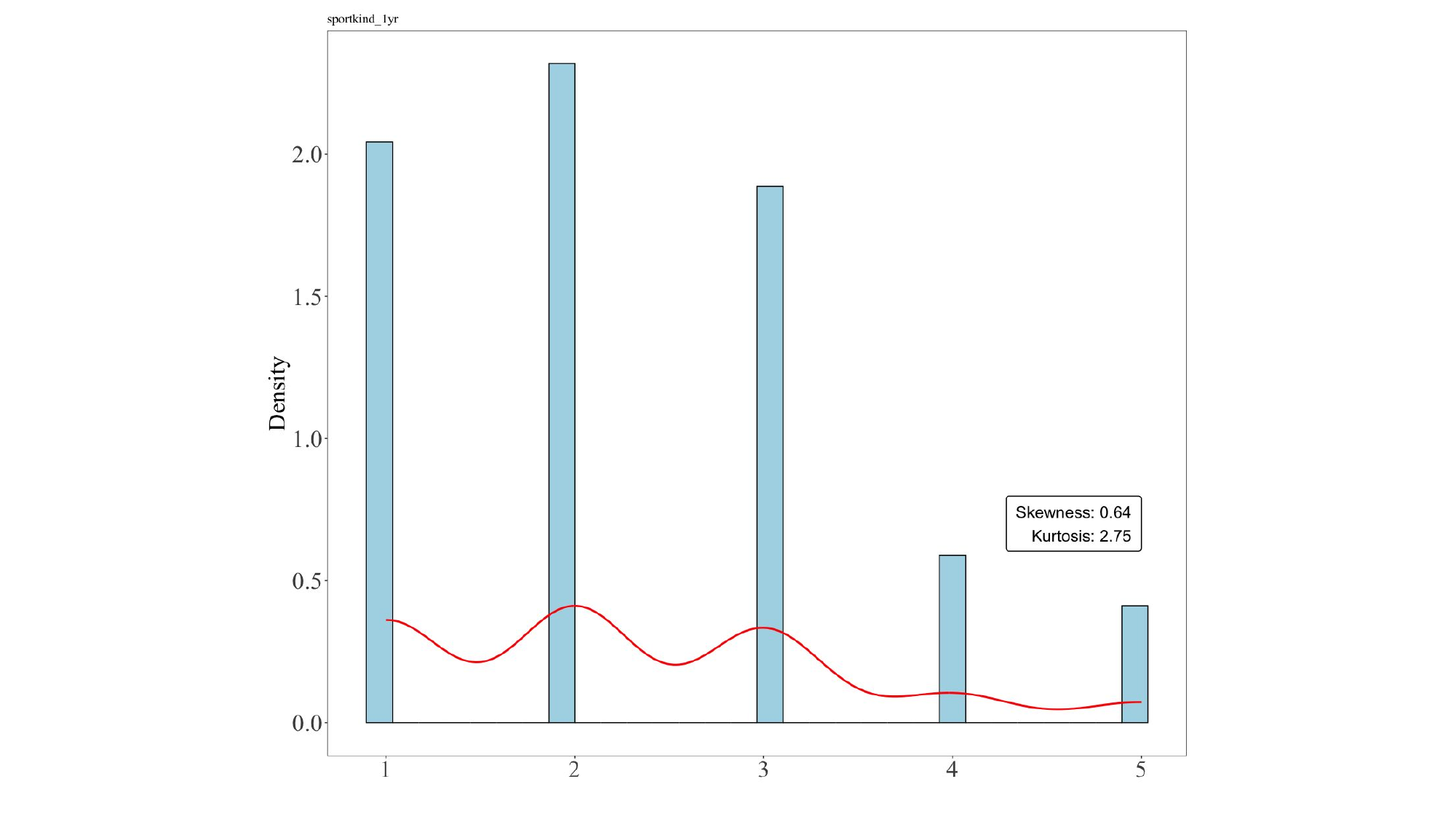

## Slide 7
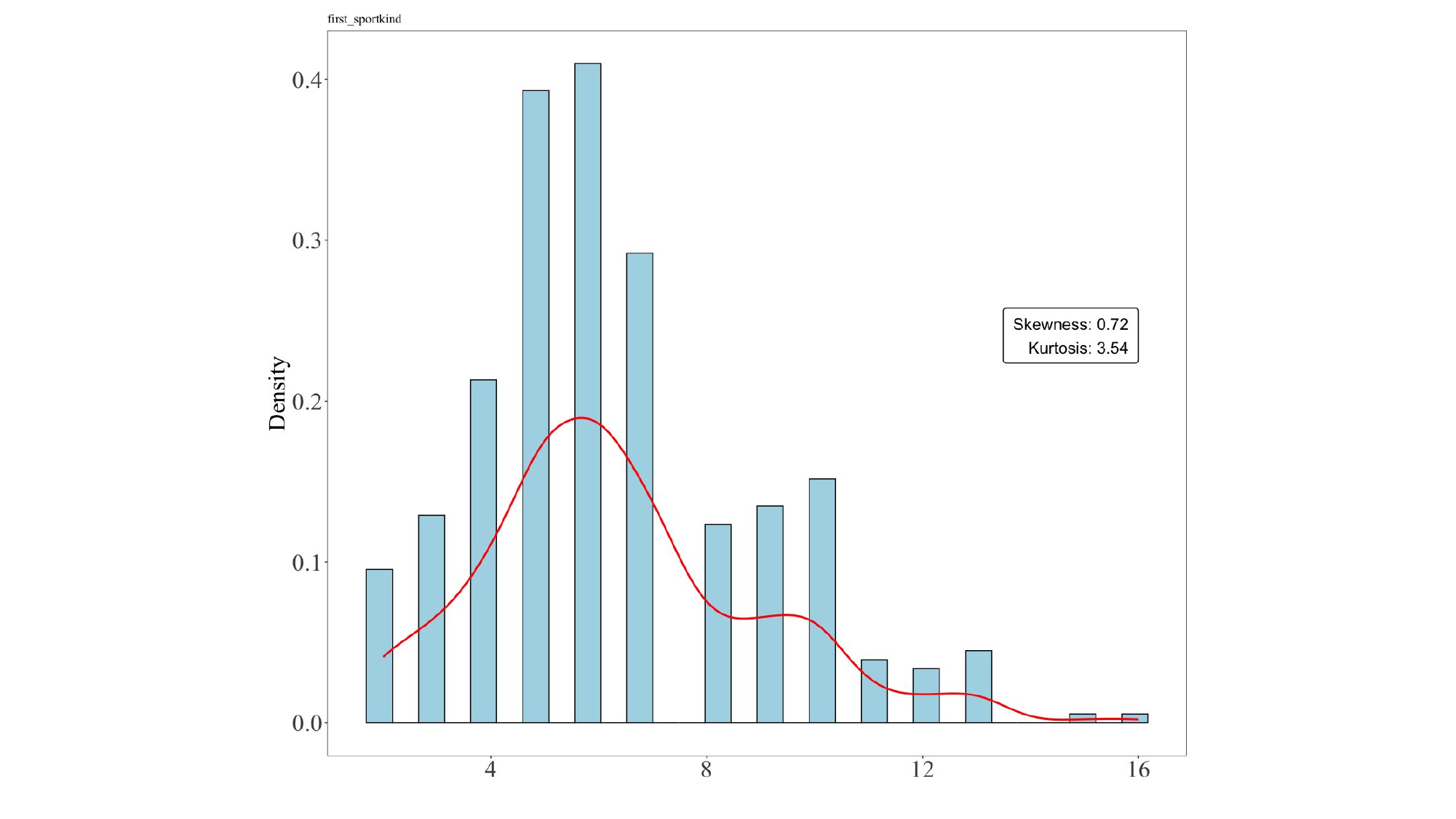

## Slide 8
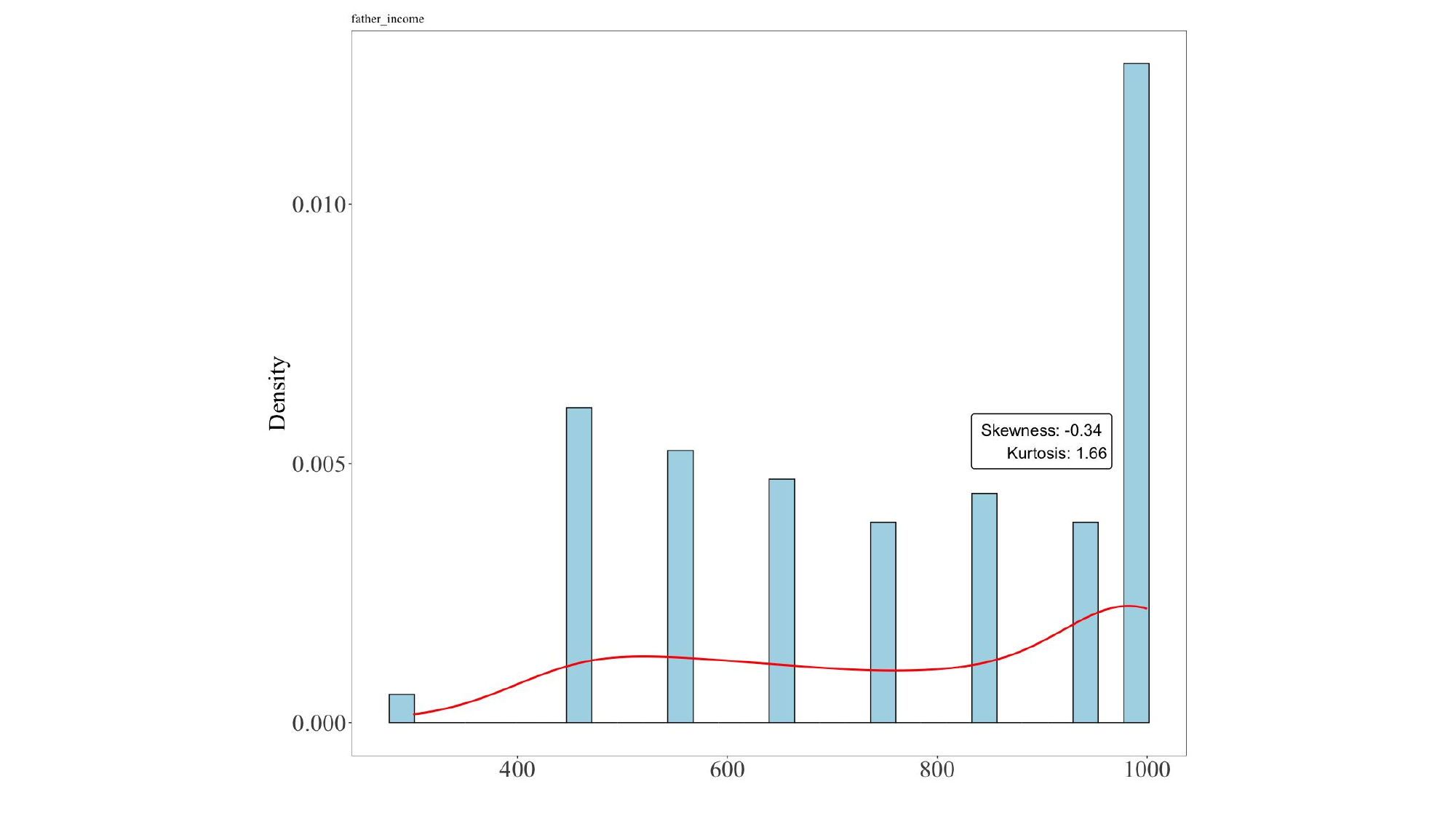

## Slide 9
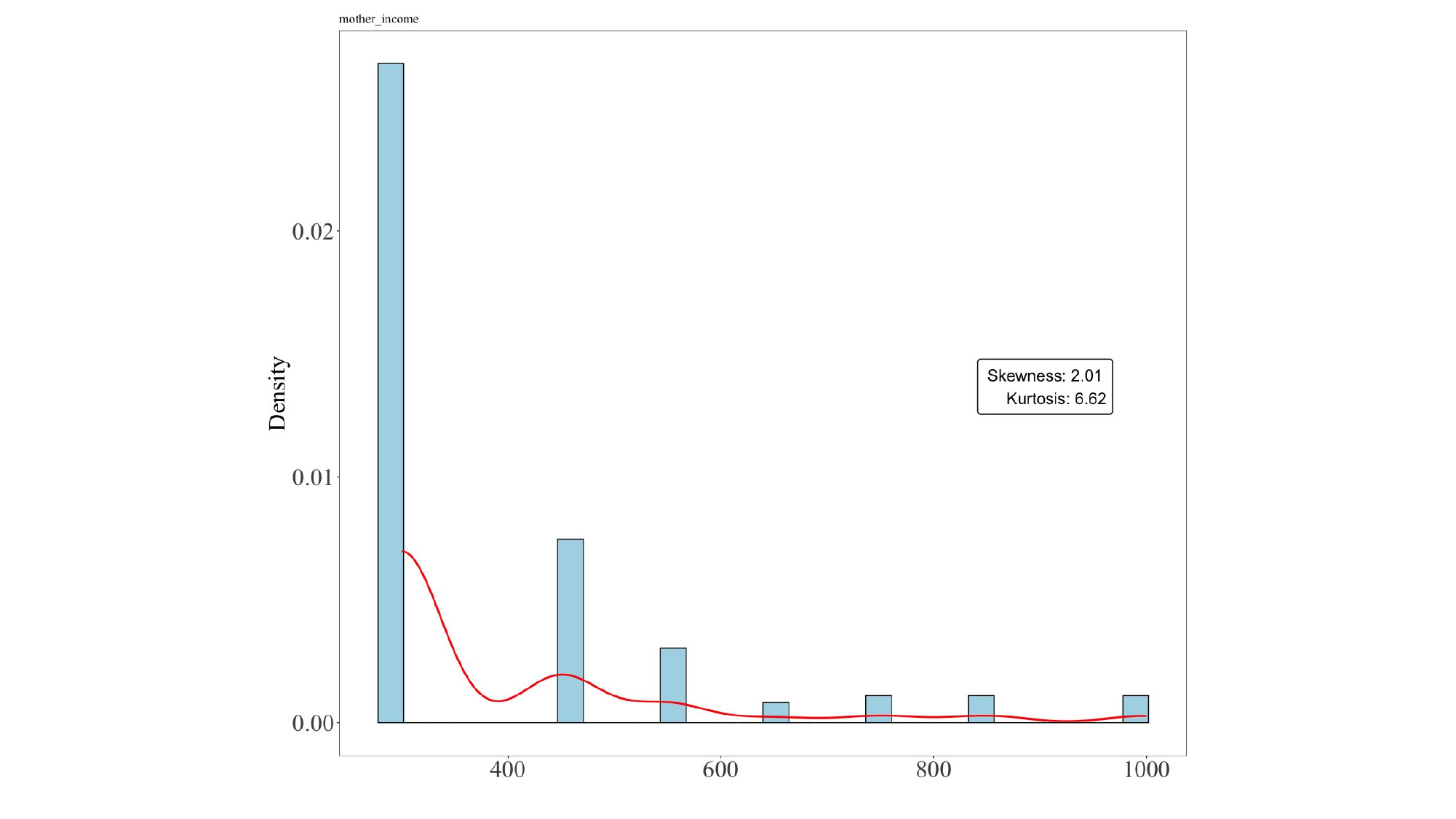

## Slide 10
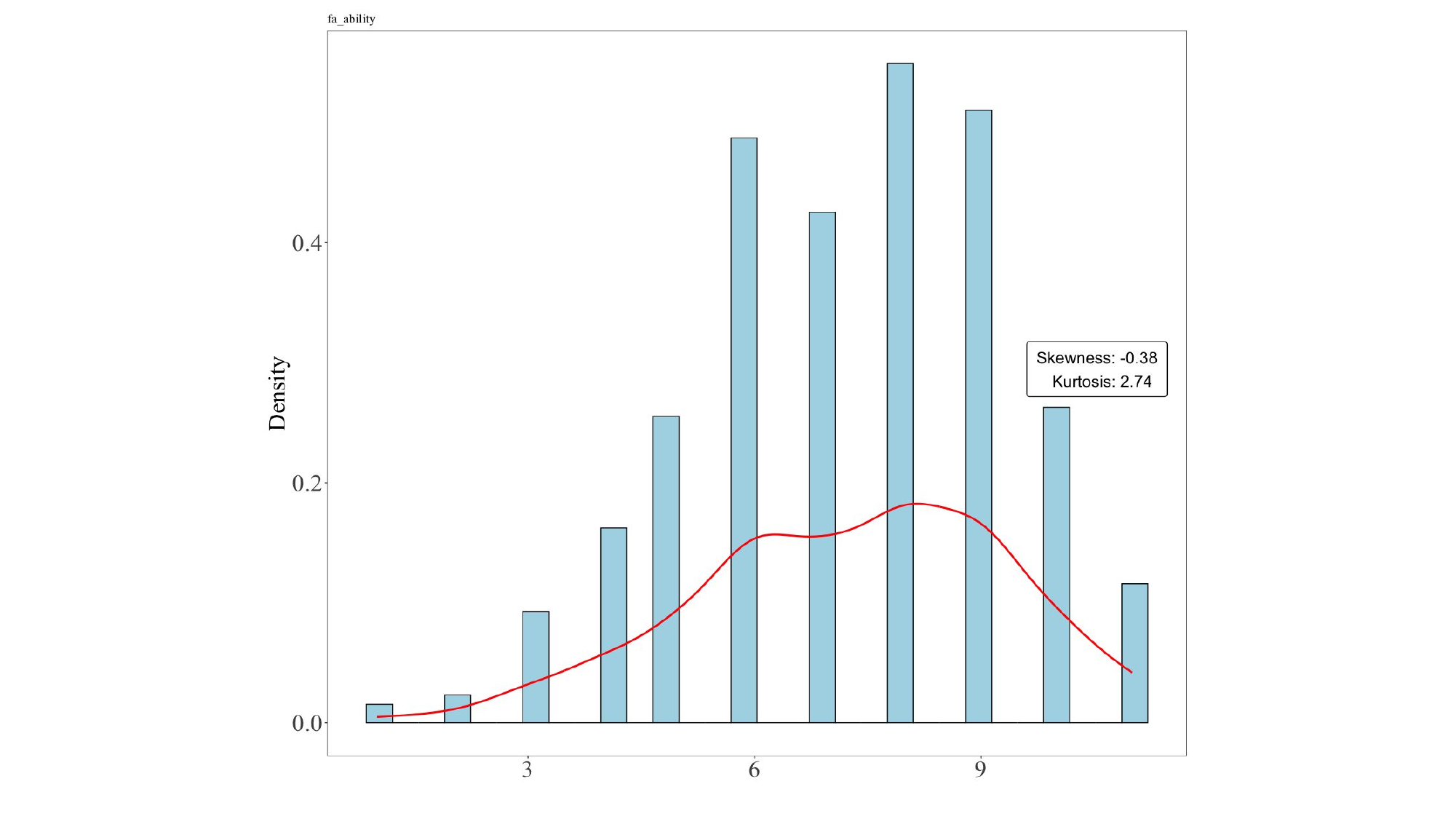

## Slide 11
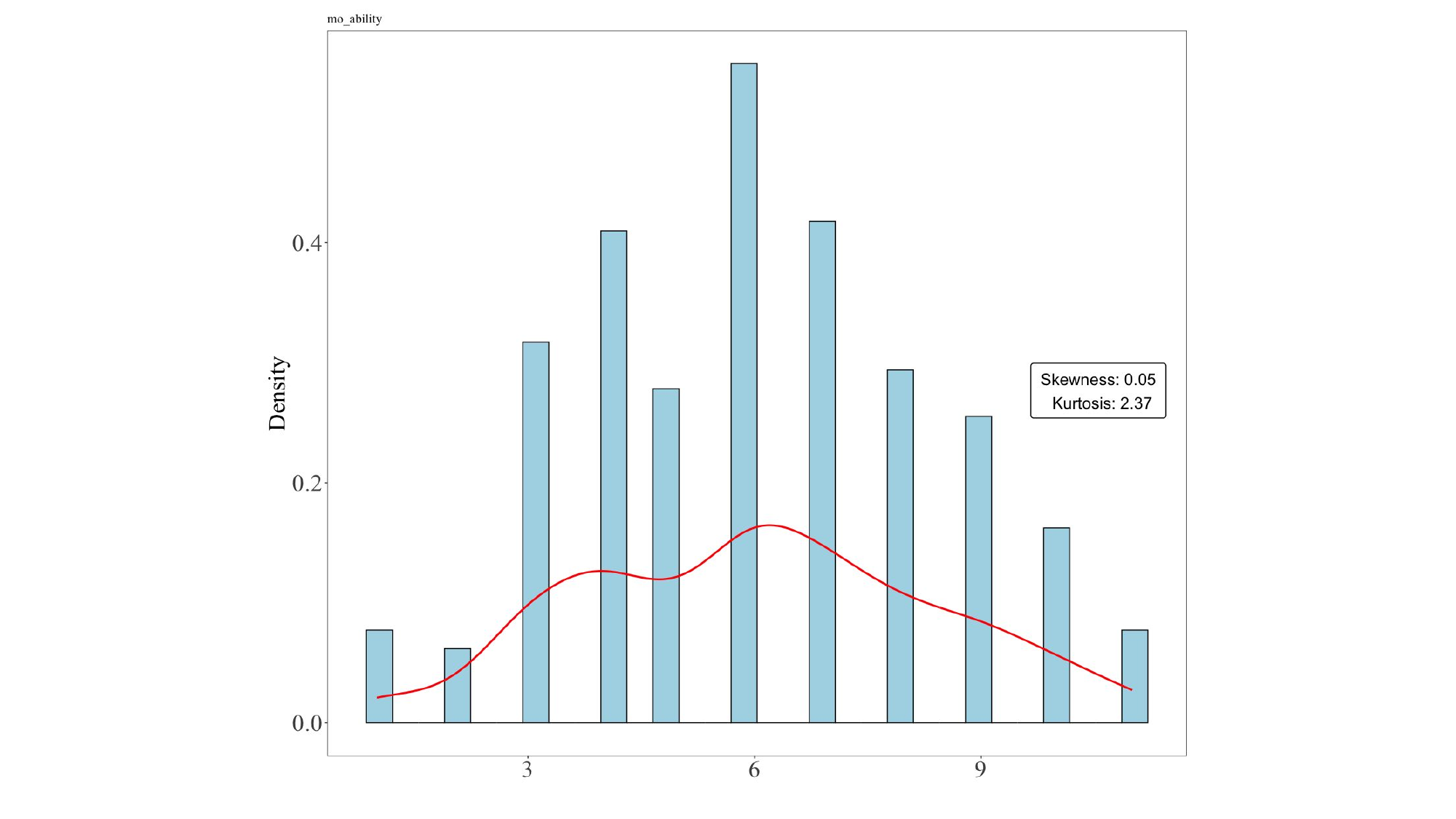

## Slide 12
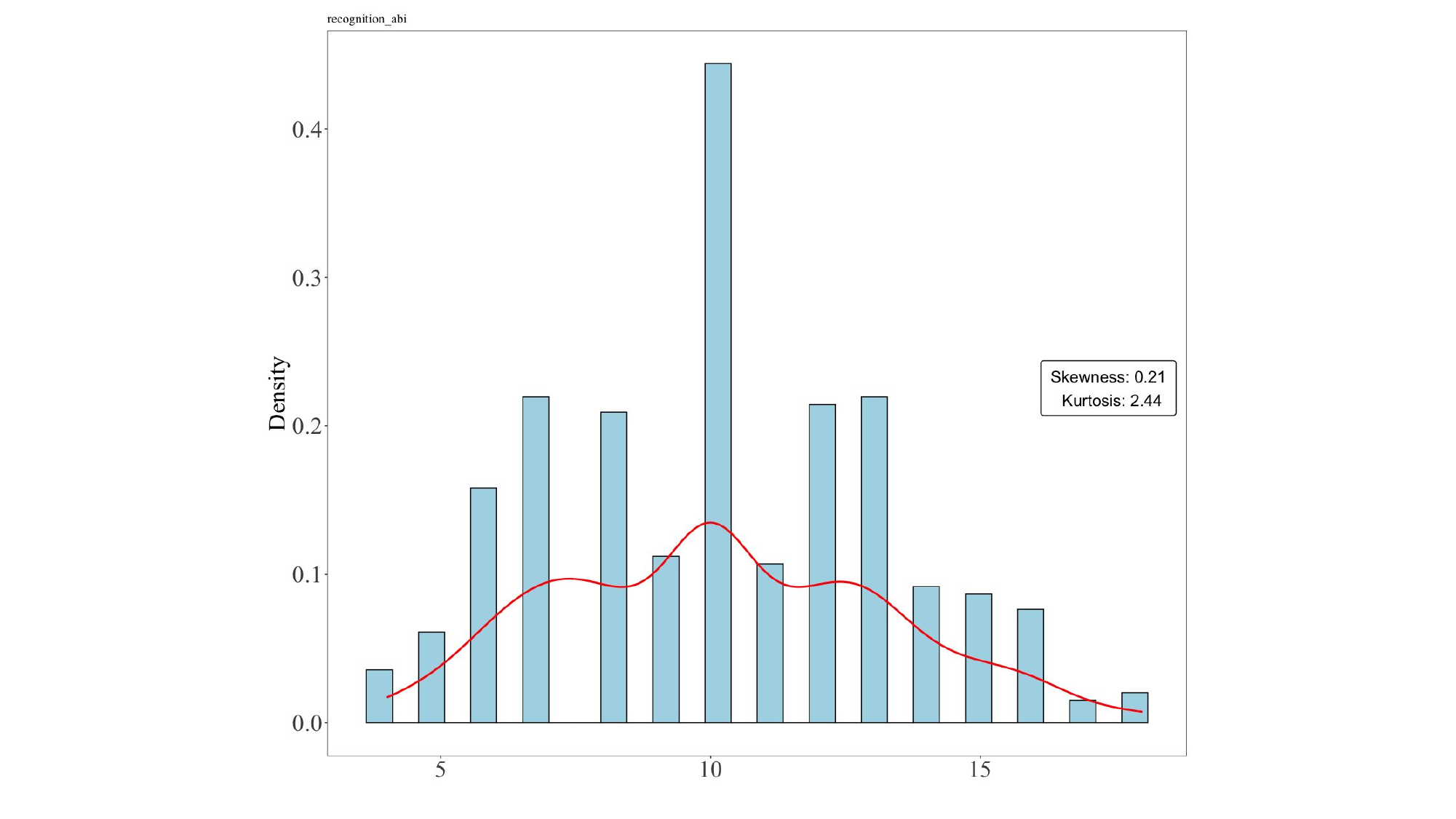

## Slide 13
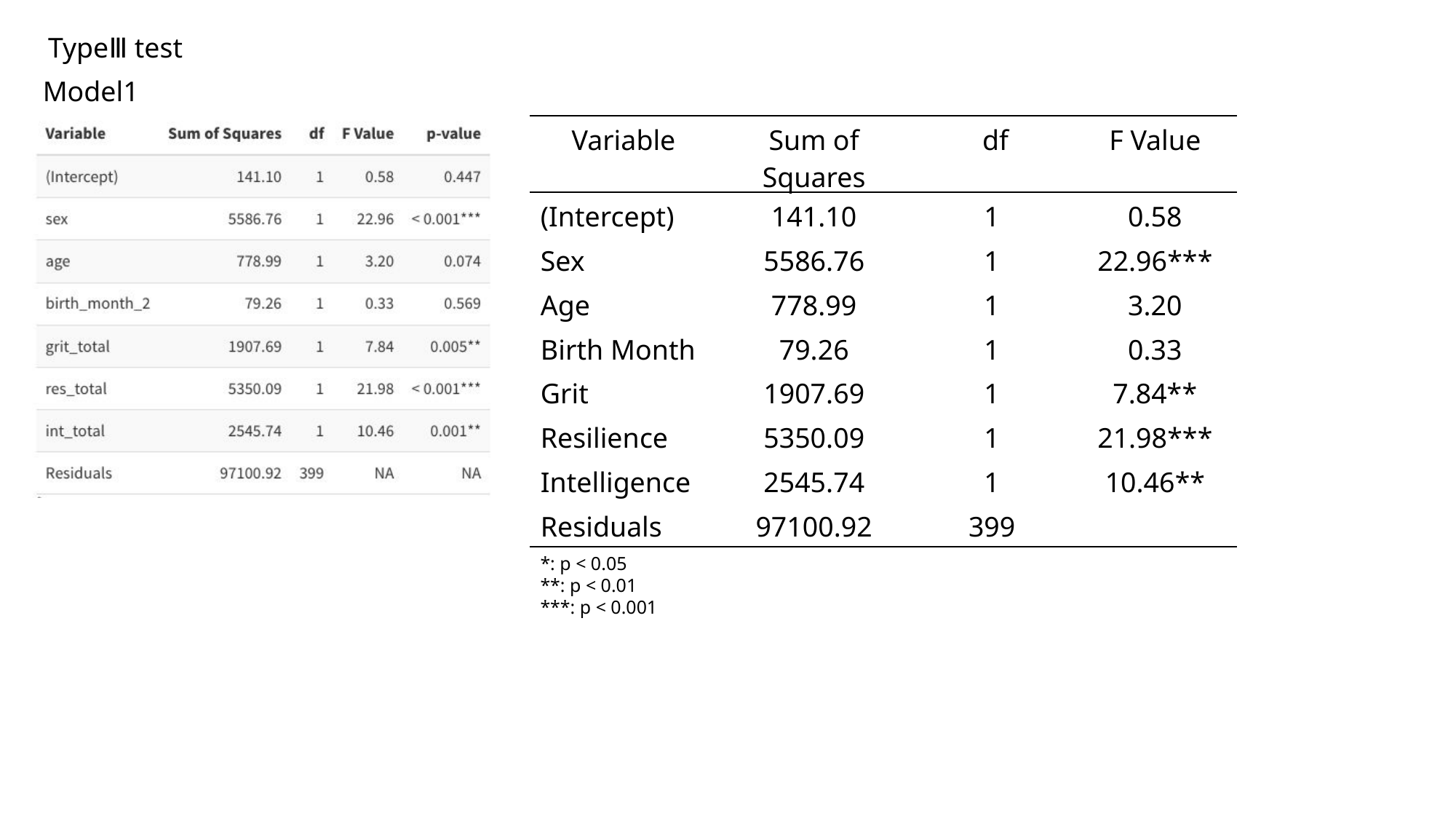

TypeⅢ test
Model1
| Variable | Sum of Squares | df | F Value |
| --- | --- | --- | --- |
| (Intercept) | 141.10 | 1 | 0.58 |
| Sex | 5586.76 | 1 | 22.96\*\*\* |
| Age | 778.99 | 1 | 3.20 |
| Birth Month | 79.26 | 1 | 0.33 |
| Grit | 1907.69 | 1 | 7.84\*\* |
| Resilience | 5350.09 | 1 | 21.98\*\*\* |
| Intelligence | 2545.74 | 1 | 10.46\*\* |
| Residuals | 97100.92 | 399 | |
*: p < 0.05
**: p < 0.01
***: p < 0.001

## Slide 14
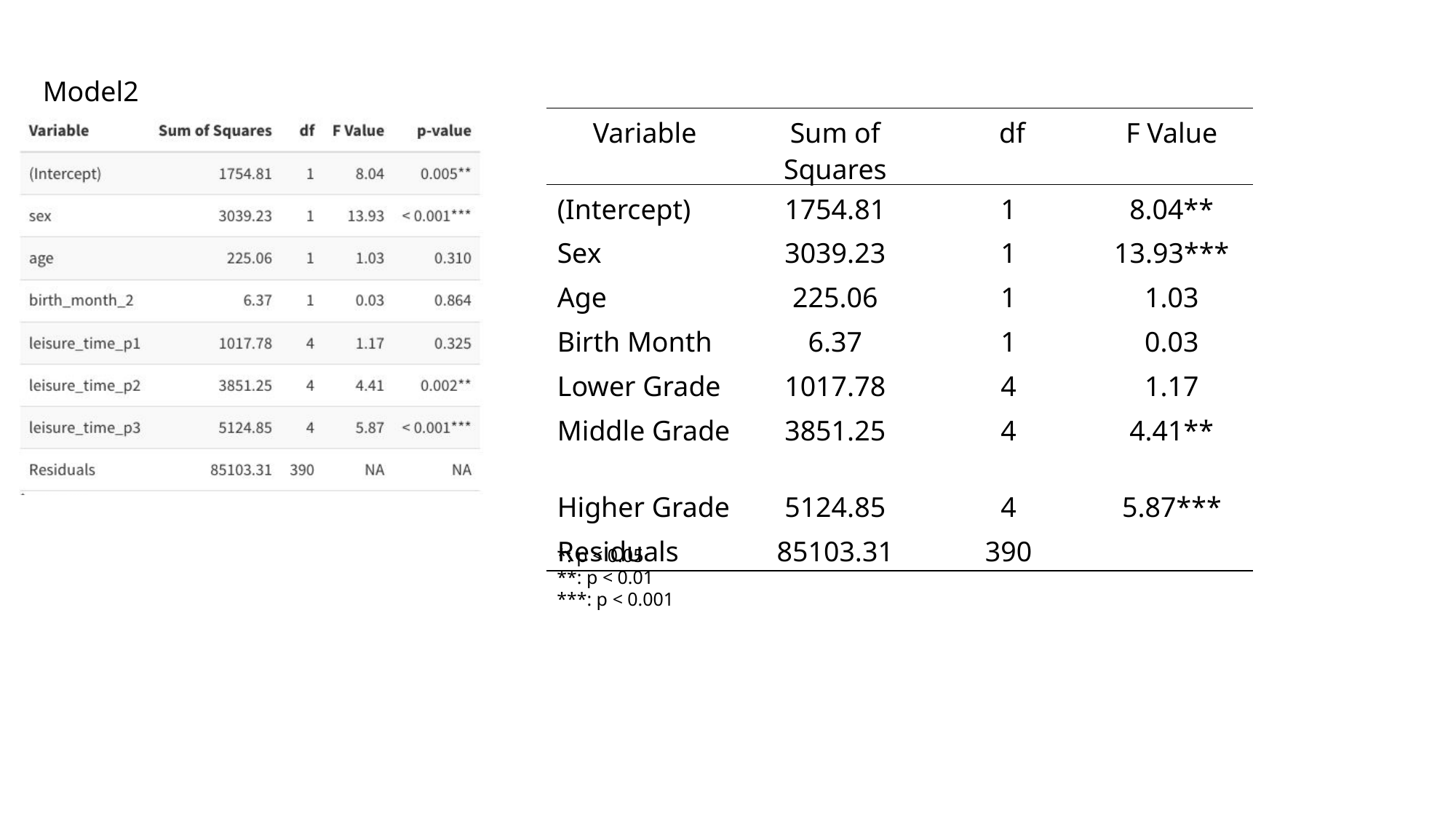

Model2
| Variable | Sum of Squares | df | F Value |
| --- | --- | --- | --- |
| (Intercept) | 1754.81 | 1 | 8.04\*\* |
| Sex | 3039.23 | 1 | 13.93\*\*\* |
| Age | 225.06 | 1 | 1.03 |
| Birth Month | 6.37 | 1 | 0.03 |
| Lower Grade | 1017.78 | 4 | 1.17 |
| Middle Grade | 3851.25 | 4 | 4.41\*\* |
| Higher Grade | 5124.85 | 4 | 5.87\*\*\* |
| Residuals | 85103.31 | 390 | |
*: p < 0.05
**: p < 0.01
***: p < 0.001

## Slide 15
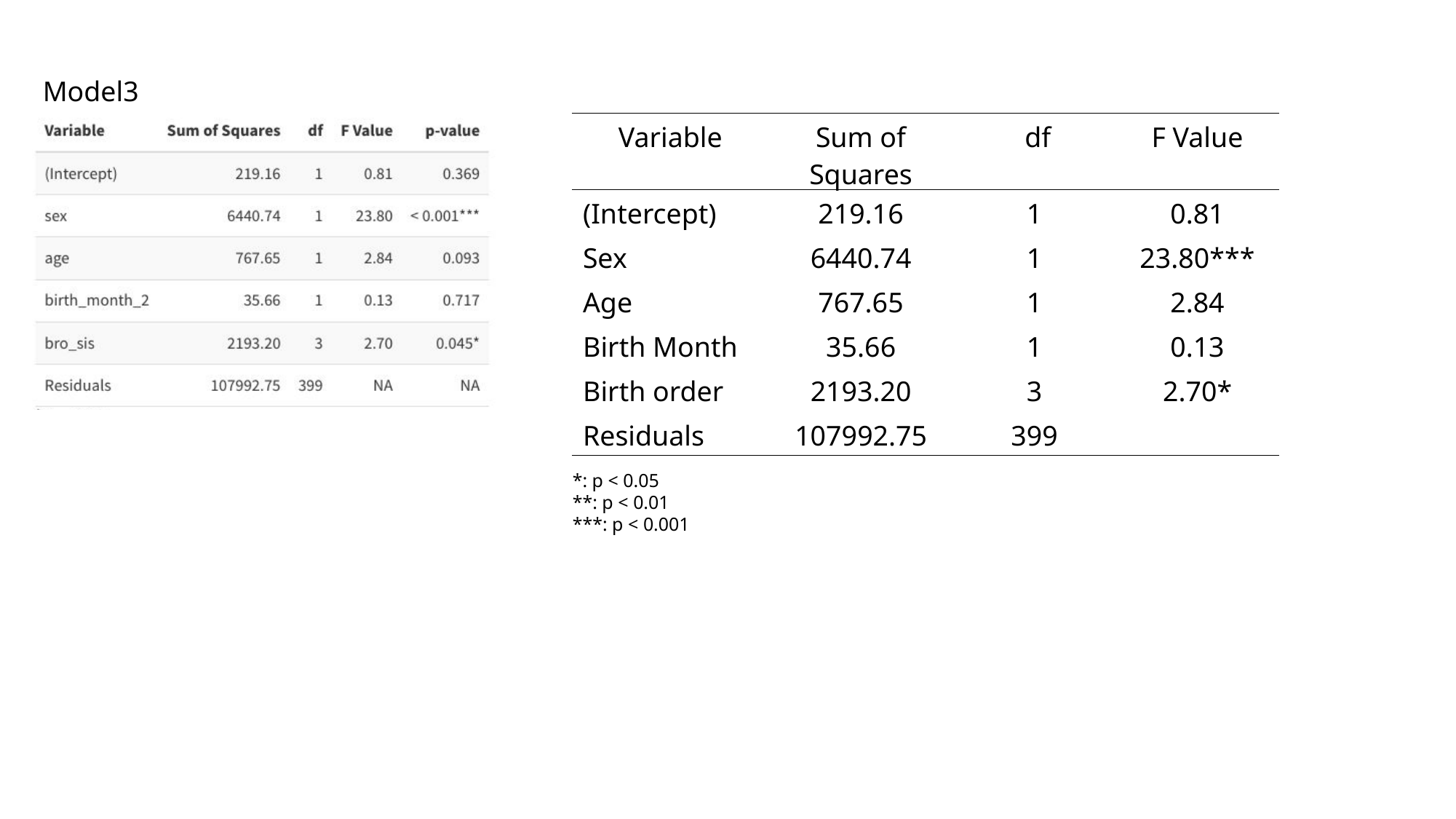

Model3
| Variable | Sum of Squares | df | F Value |
| --- | --- | --- | --- |
| (Intercept) | 219.16 | 1 | 0.81 |
| Sex | 6440.74 | 1 | 23.80\*\*\* |
| Age | 767.65 | 1 | 2.84 |
| Birth Month | 35.66 | 1 | 0.13 |
| Birth order | 2193.20 | 3 | 2.70\* |
| Residuals | 107992.75 | 399 | |
*: p < 0.05
**: p < 0.01
***: p < 0.001

## Slide 16
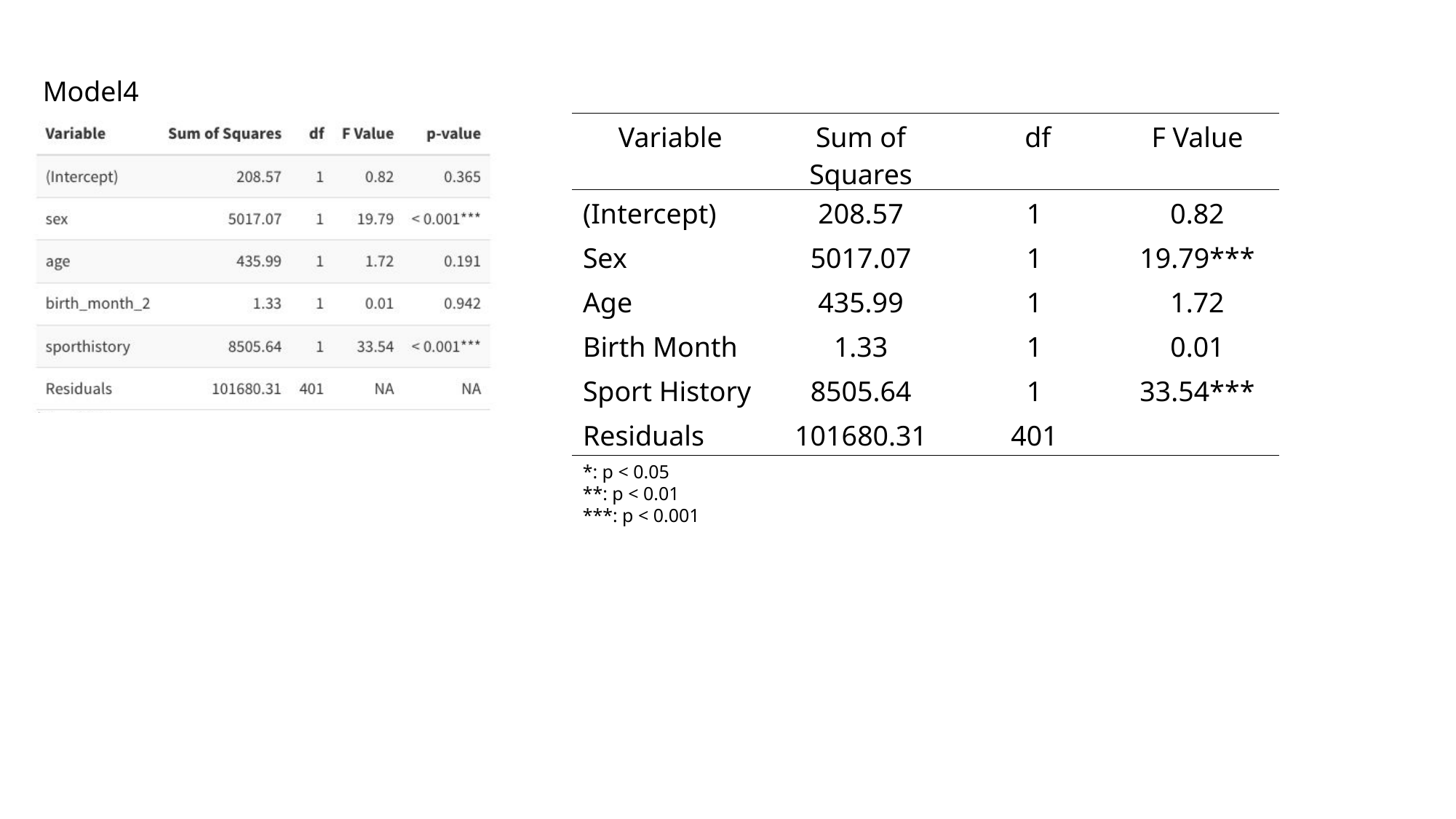

Model4
| Variable | Sum of Squares | df | F Value |
| --- | --- | --- | --- |
| (Intercept) | 208.57 | 1 | 0.82 |
| Sex | 5017.07 | 1 | 19.79\*\*\* |
| Age | 435.99 | 1 | 1.72 |
| Birth Month | 1.33 | 1 | 0.01 |
| Sport History | 8505.64 | 1 | 33.54\*\*\* |
| Residuals | 101680.31 | 401 | |
*: p < 0.05
**: p < 0.01
***: p < 0.001

## Slide 17
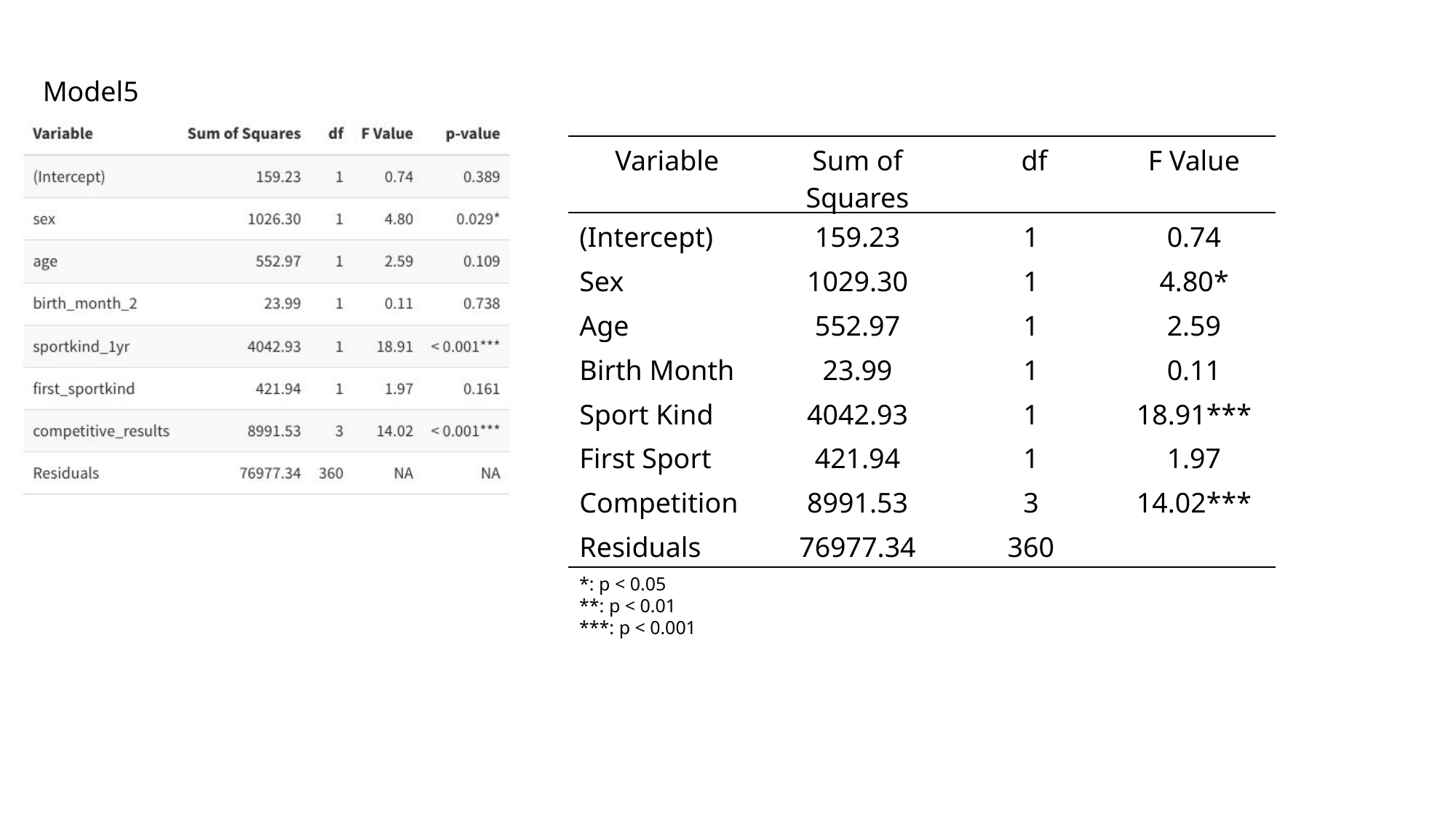

Model5
| Variable | Sum of Squares | df | F Value |
| --- | --- | --- | --- |
| (Intercept) | 159.23 | 1 | 0.74 |
| Sex | 1029.30 | 1 | 4.80\* |
| Age | 552.97 | 1 | 2.59 |
| Birth Month | 23.99 | 1 | 0.11 |
| Sport Kind | 4042.93 | 1 | 18.91\*\*\* |
| First Sport | 421.94 | 1 | 1.97 |
| Competition | 8991.53 | 3 | 14.02\*\*\* |
| Residuals | 76977.34 | 360 | |
*: p < 0.05
**: p < 0.01
***: p < 0.001

## Slide 18
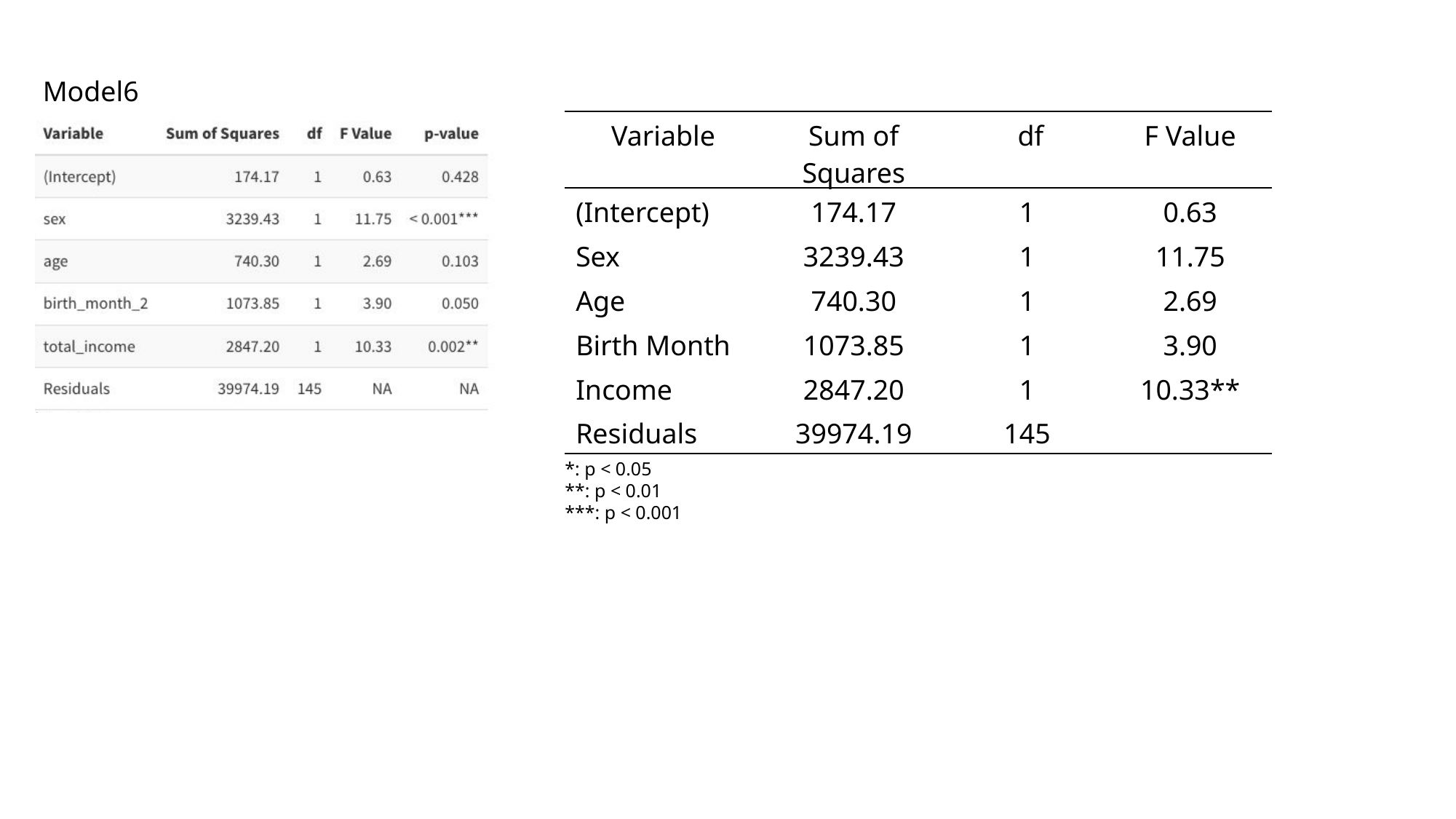

Model6
| Variable | Sum of Squares | df | F Value |
| --- | --- | --- | --- |
| (Intercept) | 174.17 | 1 | 0.63 |
| Sex | 3239.43 | 1 | 11.75 |
| Age | 740.30 | 1 | 2.69 |
| Birth Month | 1073.85 | 1 | 3.90 |
| Income | 2847.20 | 1 | 10.33\*\* |
| Residuals | 39974.19 | 145 | |
*: p < 0.05
**: p < 0.01
***: p < 0.001

## Slide 19
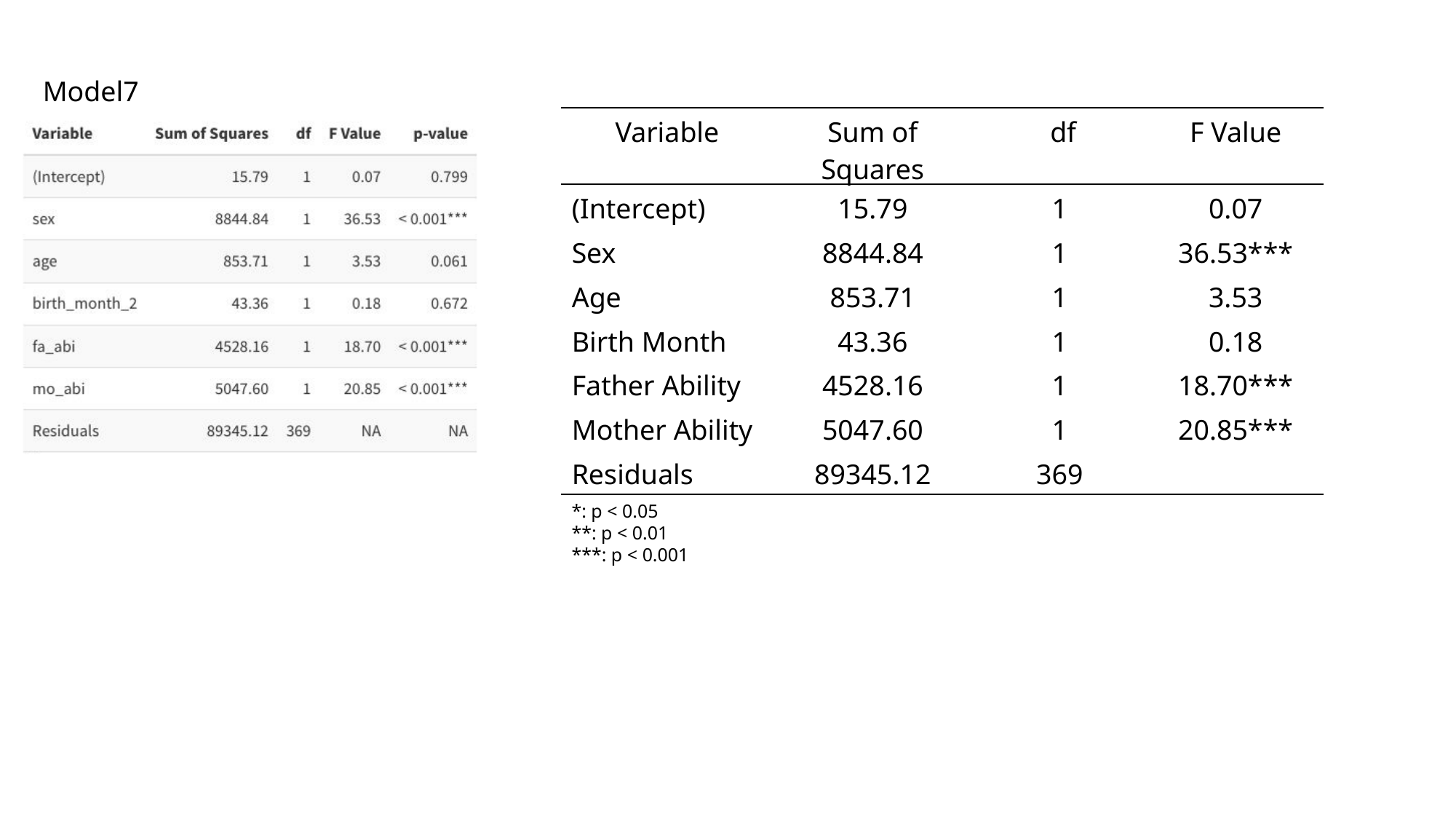

Model7
| Variable | Sum of Squares | df | F Value |
| --- | --- | --- | --- |
| (Intercept) | 15.79 | 1 | 0.07 |
| Sex | 8844.84 | 1 | 36.53\*\*\* |
| Age | 853.71 | 1 | 3.53 |
| Birth Month | 43.36 | 1 | 0.18 |
| Father Ability | 4528.16 | 1 | 18.70\*\*\* |
| Mother Ability | 5047.60 | 1 | 20.85\*\*\* |
| Residuals | 89345.12 | 369 | |
*: p < 0.05
**: p < 0.01
***: p < 0.001

## Slide 20
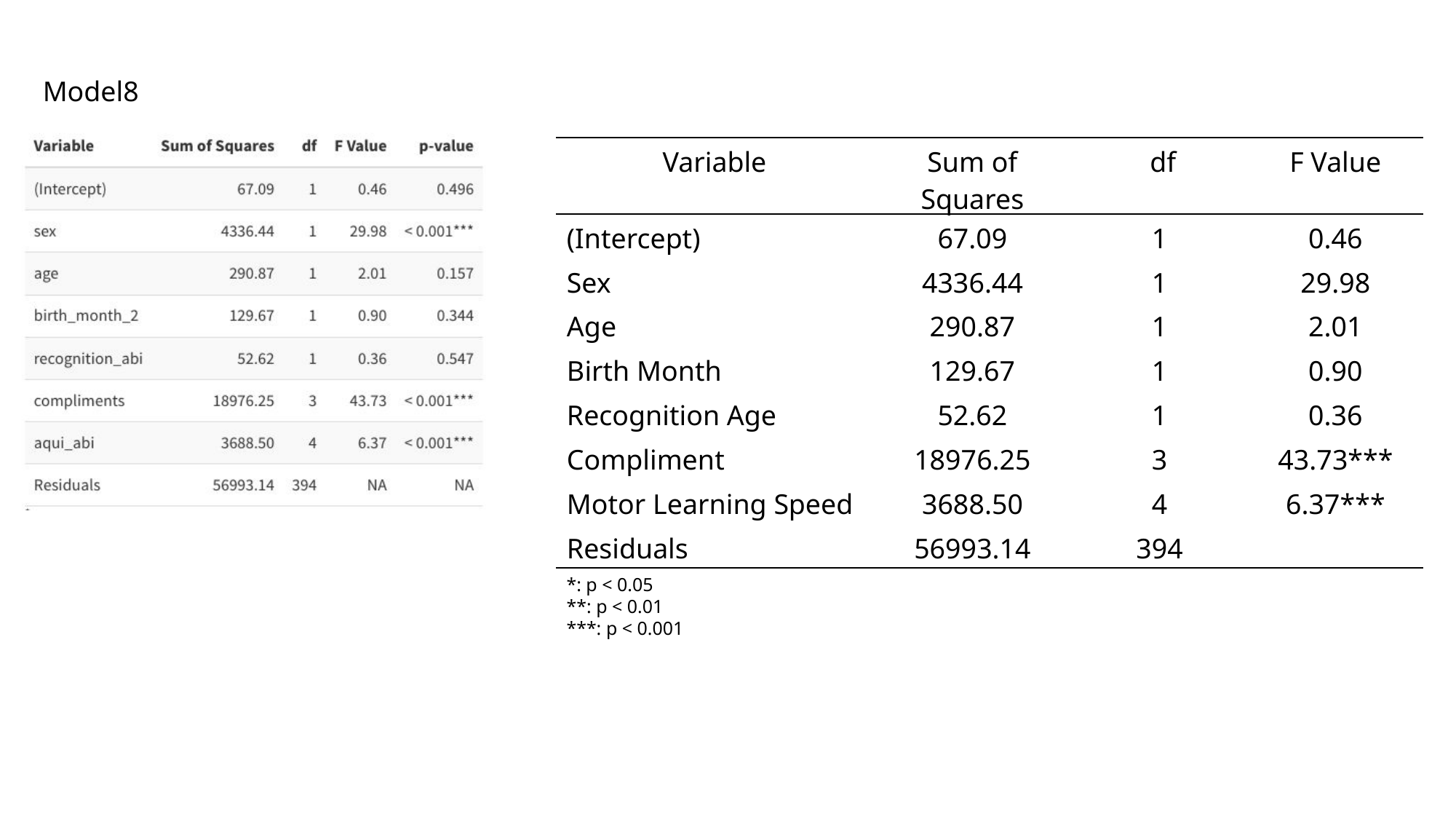

Model8
| Variable | Sum of Squares | df | F Value |
| --- | --- | --- | --- |
| (Intercept) | 67.09 | 1 | 0.46 |
| Sex | 4336.44 | 1 | 29.98 |
| Age | 290.87 | 1 | 2.01 |
| Birth Month | 129.67 | 1 | 0.90 |
| Recognition Age | 52.62 | 1 | 0.36 |
| Compliment | 18976.25 | 3 | 43.73\*\*\* |
| Motor Learning Speed | 3688.50 | 4 | 6.37\*\*\* |
| Residuals | 56993.14 | 394 | |
*: p < 0.05
**: p < 0.01
***: p < 0.001
