# Supplementary material for: Determinants of subjective total athletic ability
Source: PLoS One. 2025 May 28;20(5):e0324044. doi: 10.1371/journal.pone.0324044 (PMC12118886; doi:10.1371/journal.pone.0324044)
Supplement: Attachment — Submitted filename: PONE-D-24-20019_reviewer.pdf [file pone.0324044.s004.pdf]

PLOS ONE

Determinants of subjective total athletic ability

--Manuscript Draft--

**Manuscript Number:**	PONE-D-24-20019
**Article Type:**	Research Article
**Full Title:**	Determinants of subjective total athletic ability
**Short Title:**	Factors affecting motor skills
**Corresponding Author:**	Sho Ito, Ph.D.<br>Nanzan University: Nanzan Daigaku<br>Nagoya, JAPAN
**Keywords:**	athletic anility; college students; Personality traits; sibling composition; parents' athletic ability
**Abstract:**	The term "good motor skill" is often discussed in everyday contexts and when observing sports; however, its definition remains elusive, and the associated factors are not well understood. Therefore, in this cross-sectional study, we investigated the determinants of subjective total athletic ability. A cohort of 406 graduate students completed a questionnaire to evaluate their perceived athletic prowess in various sports, and assessments of their personality traits, family background, and sports performance. The analysis revealed correlations between the perceived general athletic abilities and specific abilities in soccer, volleyball, basketball, and short-distance racing. Furthermore, linear model analyses indicated a positive association between perceived total athletic abilities and personal characteristics such as grit, resilience, and a growth mindset. Factors such as recreational activities in elementary school, sibling structure, prior athletic experience, parental athletic ability, family income, external evaluations of motor skills, and age at first walking also appeared to influence perceived athletic ability. These results imply that a blend of internal and external factors may shape subjective athletic ability. However, future studies should investigate the causal connections among these factors to deepen our understanding of the concept and its influencers.
**Order of Authors:**	Sho Ito, Ph.D.
	Keishi Soga, Ph.D.
	Kouki Kato, Ph.D.
**Additional Information:**	
**Question**	**Response**
**Financial Disclosure**<br><br>Enter a financial disclosure statement that describes the sources of funding for the work included in this submission. Review the submission guidelines for detailed requirements. View published research articles from *PLOS ONE* for specific examples.<br><br>This statement is required for submission and **will appear in the published article** if the submission is accepted. Please make sure it is accurate.	Yes

Powered by Editorial Manager® and ProduXion Manager® from Aries Systems Corporation

<table>
<tr><td>Funded studies
Enter a statement with the following details:
• Initials of the authors who received each award
• Grant numbers awarded to each author
• The full name of each funder
• URL of each funder website
• Did the sponsors or funders play any role in the study design, data collection and analysis, decision to publish, or preparation of the manuscript?

Did you receive funding for this work?</td><td></td></tr>
<tr><td>Please add funding details.
as follow-up to "Financial Disclosure

Enter a financial disclosure statement that describes the sources of funding for the work included in this submission. Review the submission guidelines for detailed requirements. View published research articles from PLOS ONE for specific examples.

This statement is required for submission and will appear in the published article if the submission is accepted. Please make sure it is accurate.

Funded studies
Enter a statement with the following details:
• Initials of the authors who received each award
• Grant numbers awarded to each author
• The full name of each funder
• URL of each funder website
• Did the sponsors or funders play any role in the study design, data collection and analysis, decision to publish, or preparation of the manuscript?

Did you receive funding for this work?"</td><td>We would like to express our sincere gratitude to the Nanzan University Pache Research Subsidy I-A-2 for the 2024 academic year and JSPS KAKENHI Grant Number JP23K10623 for funding this research.
1
Initials of the authors who received each award; Kouki Kato
Grant numbers awarded to each author ; JP23K10623
The full name of each funder: Japan Society for the Promotion of Science
URL of each funder website; https://www.jsps.go.jp/j-grantsinaid/
Did the sponsors or funders play any role in the study design, data collection and analysis, decision to publish, or preparation of the manuscript?;No
2
Initials of the authors who received each award; Kouki Kato
Grant numbers awarded to each author ; Nanzan University Pache Research Subsidy I-A-2 for the 2024 academic year
The full name of each funder: Nanzan University
URL of each funder website; https://www.nanzan-u.ac.jp/
Did the sponsors or funders play any role in the study design, data collection and analysis, decision to publish, or preparation of the manuscript?;No</td></tr>
<tr><td>Please select the country of your main research funder (please select carefully as in some cases this is used in fee calculation).</td><td>JAPAN - JP</td></tr>
</table>

as follow-up to "**Financial Disclosure**<br><br>Enter a financial disclosure statement that describes the sources of funding for the work included in this submission. Review the submission guidelines for detailed requirements. View published research articles from *PLOS ONE* for specific examples.<br><br>This statement is required for submission and **will appear in the published article** if the submission is accepted. Please make sure it is accurate.<br><br>**Funded studies**<br>Enter a statement with the following details:<br>• Initials of the authors who received each award<br>• Grant numbers awarded to each author<br>• The full name of each funder<br>• URL of each funder website<br>• Did the sponsors or funders play any role in the study design, data collection and analysis, decision to publish, or preparation of the manuscript?<br><br>Did you receive funding for this work?"	
**Competing Interests**<br><br>Use the instructions below to enter a competing interest statement for this submission. On behalf of all authors, disclose any competing interests that could be perceived to bias this work—acknowledging all financial support and any other relevant financial or non-financial competing interests.<br><br>This statement is required for submission and **will appear in the published article** if the submission is accepted. Please make sure it is accurate and that any funding sources listed in your Funding Information later in the submission form are also declared in your Financial Disclosure statement.	The authors have declared that no competing interests exist.

Powered by Editorial Manager® and ProduXion Manager® from Aries Systems Corporation

View published research articles from *PLOS ONE* for specific examples.<br><br>**NO authors have competing interests**<br><br>Enter: *The authors have declared that no competing interests exist.*<br><br>**Authors with competing interests**<br><br>Enter competing interest details beginning with this statement:<br><br>*I have read the journal's policy and the authors of this manuscript have the following competing interests: [insert competing interests here]*<br><br>* typeset	
**Ethics Statement**<br><br>Enter an ethics statement for this submission. This statement is required if the study involved:<br><br>• Human participants<br>• Human specimens or tissue<br>• Vertebrate animals or cephalopods<br>• Vertebrate embryos or tissues<br>• Field research<br><br>Write "N/A" if the submission does not require an ethics statement.<br><br>General guidance is provided below. Consult the submission guidelines for detailed instructions. **Make sure that all information entered here is included in the Methods section of the manuscript.**	This study was approved by the Ethics Committee for Human Subjects of Nanzan University (Approval No. 23-072). In this study, informed consent was obtained from participants through Google Forms (Alphabet Inc., U.S.A.). Informed consent for participation in the study was waived by the ethics committee, including the consent from parents or guardians of the minors involved. The data for this study were collected between December 5, 2023, and December 18, 2023.

Powered by Editorial Manager® and ProduXion Manager® from Aries Systems Corporation

**Format for specific study types**<br><br>**Human Subject Research (involving human participants and/or tissue)**<br>• Give the name of the institutional review board or ethics committee that approved the study<br>• Include the approval number and/or a statement indicating approval of this research<br>• Indicate the form of consent obtained (written/oral) or the reason that consent was not obtained (e.g. the data were analyzed anonymously)<br><br>**Animal Research (involving vertebrate animals, embryos or tissues)**<br>• Provide the name of the Institutional Animal Care and Use Committee (IACUC) or other relevant ethics board that reviewed the study protocol, and indicate whether they approved this research or granted a formal waiver of ethical approval<br>• Include an approval number if one was obtained<br>• If the study involved *non-human primates*, add *additional details* about animal welfare and steps taken to ameliorate suffering<br>• If anesthesia, euthanasia, or any kind of animal sacrifice is part of the study, include briefly which substances and/or methods were applied<br><br>**Field Research**<br><br>Include the following details if this study involves the collection of plant, animal, or other materials from a natural setting:<br>• Field permit number<br>• Name of the institution or relevant body that granted permission	
**Data Availability**<br><br>Authors are required to make all data underlying the findings described fully available, without restriction, and from the time of publication. PLOS allows rare exceptions to address legal and ethical concerns. See the PLOS Data Policy and FAQ for detailed information.	Yes - all data are fully available without restriction

A Data Availability Statement describing where the data can be found is required at submission. Your answers to this question constitute the Data Availability Statement and **will be published in the article**, if accepted.<br><br>**Important:** Stating 'data available on request from the author' is not sufficient. If your data are only available upon request, select 'No' for the first question and explain your exceptional situation in the text box.<br><br>Do the authors confirm that all data underlying the findings described in their manuscript are fully available without restriction?	
**Describe where the data may be found in full sentences. If you are copying our sample text, replace any instances of XXX with the appropriate details.**<br><br>• If the data are **held or will be held in a public repository**, include URLs, accession numbers or DOIs. If this information will only be available after acceptance, indicate this by ticking the box below. For example: *All XXX files are available from the XXX database (accession number(s) XXX, XXX.*).<br>• If the data are all contained **within the manuscript and/or Supporting Information files**, enter the following: *All relevant data are within the manuscript and its Supporting Information files.*<br>• If neither of these applies but you are able to provide **details of access elsewhere**, with or without limitations, please do so. For example:<br><br>*Data cannot be shared publicly because of [XXX]. Data are available from the XXX Institutional Data Access / Ethics Committee (contact via XXX) for researchers who meet the criteria for access to confidential data.*<br><br>*The data underlying the results presented in the study are available from (include the name of the third party*	All relevant data are within the manuscript and its Supporting Information files.

and contact information or URL).<br>• This text is appropriate if the data are owned by a third party and authors do not have permission to share the data.<br><br>* typeset	
Additional data availability information:	

Manuscript

Click here to access/download;Manuscript;The_factors_involved_in_gross_

Determinants of subjective total athletic ability

Short title: Factors affecting motor skills

Sho Ito[1*], Keishi Soga[2], Kouki Kato[1]

[1] Physical Education Center, Nanzan University, Nagoya, Aichi, Japan

[2] Smart Aging International Research Center, Tohoku University, Sendai, Miyagi, Japan

* Corresponding author

Sho Ito

E-mail：shoito@nanzan-u.ac.jp

Abstract

The term "good motor skill" is often discussed in everyday contexts and when observing sports; however, its definition remains elusive, and the associated factors are not well understood. Therefore, in this cross-sectional study, we investigated the determinants of subjective total athletic ability. A cohort of 406 graduate students completed a questionnaire to evaluate their perceived athletic prowess in various sports, and assessments of their personality traits, family background, and sports performance. The analysis revealed correlations between the perceived general athletic abilities and specific abilities in soccer, volleyball, basketball, and short-distance racing. Furthermore, linear model analyses indicated a positive association between perceived total athletic abilities and personal characteristics such as grit, resilience, and a growth mindset. Factors such as recreational activities in elementary school, sibling structure, prior athletic experience, parental athletic ability, family income, external evaluations of motor skills, and age at first walking also appeared to influence perceived athletic ability. These results imply that a blend of internal and external factors may shape subjective athletic ability. However, future studies should investigate the causal connections among these factors to deepen our understanding of the concept and its influencers.

Introduction

The awareness of subjective athletic ability is crucial for an individual's participation in physical activities and sports competitions. A strong perception of athletic competence fosters engagement, whereas feeling inferior in skills may discourage participation. Participation in sports and physical activities offers numerous health benefits [1] and can considerably influence students's academic records [2]. Despite frequent references to "He/She is athletic" in daily conversations and sports viewership, the definition is still vague. This term is commonly understood as an "overall evaluation of physical strength, athletic capability" and the "ability to excel across various sports." Praise for athletic ability generally indicates a superior sports performance. However, the elements that contribute to motor skill proficiency are not clearly defined. Therefore, elucidating the fundamental aspects that influence motor-skill development will help establish more precise criteria for this term.

In physical education and sports psychology, research on "motor competence" has been widely conducted on children and adolescents regarding athletic ability and its evaluation [3–7]. Motor competence is a measure of self-perception of competence or confidence in physical activity [8]. Confidence resulting from successful exercise experiences (a sense of motor competence) is crucial for further improvement of athletic ability and personality development [9].

Studies have also been conducted on the relationship between lifestyle, family environment, athletic ability, and exercise habits [10–15]. These studies suggest that motor competence and athletic ability are influenced by various lifestyle factors. Notably, numerous studies have focused on children and adolescents; however, there is a notable gap in research concerning college students, who are at a critical juncture in their lives

where they begin to form lifestyles and exercise habits. In addition, most previous studies focused on physical fitness tests and specific movements [16–18], with no studies having explored abilities in various sports disciplines.

Relative age can influence physical fitness factors and characteristics in different sports [19]. These effects suggest that differences in physical and mental development among students in the same grade affect academic achievement [20] and athletic performance [19]. The relative age effect is prominent in major sports such as baseball and soccer, whereas no effects were observed for relatively minor sports such as handball and badminton [21]. Additionally, the nature of the sport, whether it is a ball game, team sport, or individual sport, may also affect subjective athletic ability. Therefore, differences in sports experience may affect a sense of motor competence. Furthermore, among junior high school students, those with higher physical fitness and motor skills tend to have a higher "perception of physical competence" in motor competence [4], suggesting that differences in sports experience can influence subjective overall athletic ability.

Successful sports experiences require perseverance, as athletes often encounter failures and setbacks along the way. Personality traits are vital for good athletic performance in sports [22]. The personality trait, grit, which is the ability to persevere, is often used as an indicator of the capacity to achieve long-term goals despite difficult situations [23]. Athletes with higher grit ability tend to spend more time actively responding to competition, leading to improvements in sports skills [24]. Additionally, resilience and a growth mindset are crucial in overcoming challenges. Resilience is the ability to bounce back from difficult situations [25], and a growth mindset is the belief in changeable talent with consistent efforts to improve one's ability [26]. High competencies in personality traits may be associated with good sports performance, leading to increased athletic

ability and motor competence.

Therefore, in this study, we aimed to conduct a questionnaire survey among college students to determine the relationship between their subjective general athletic ability (commonly referred to as motor skills) and their subjective athletic ability in various sports, personality traits, sibling composition, socio-economic status, and athletic performance.

Materials and methods

1. Participants

Data were collected from 421 survey participants who were students at Nanzan University and their acquaintances. The study's outline and purpose were fully explained to the participants, and consent was obtained from 409 participants. To consider the influence of sex in this study, 406 of those who gave consent and whose sex was specified were included in the analysis (mean age: 19.31 ± 0.95 years; female: 221, male: 185). This study was approved by the Ethics Committee for Human Subjects of Nanzan University (Approval No. 23-072). In this study, informed consent was obtained from participants through Google Forms (Alphabet Inc., U.S.A.). Informed consent for participation in the study was waived by the ethics committee, including the consent from parents or guardians of the minors involved. The data for this study were collected between December 5, 2023, and December 18, 2023.

2. Questionnaire

1) Face sheet

The respondents were asked to choose from a list of options for age, birth month, and

sex. Regarding sibling composition, participants were asked to select all that apply from the following groups: "I have an older brother within 2 years," "I have an older brother of ≥ 3 years," "I have an older sister within 2 years," "I have an older sister of ≥ 3 years," "I have a younger brother within 2 years," "I have a younger brother of ≥ 3 years," "I have a younger sister within 2 years," "I have a younger sister of ≥ 3 years," and "I am an only child." The participants were asked to select their father's and mother's annual income from the following options: "don't know," "< 3 million yen," "3.01 million – 4 million yen," "4.01 million – 5 million yen," "5.01 million – 6 million yen," "6.01 million – 7 million yen," "7.01 million – 8 million yen," "8.01 million – 9 million yen," "9.01 million – 10 million yen," and " ≥10.01 million yen ", respectively. The participants were asked to choose the age at which they were able to walk from "< 9 months," "9 months," "10 months," "11 months," "12 months," "13 months," "14 months," "15 months," "16 months," " ≥17 months," "don't know specifically, but earlier"; "don't know specifically, but about average"; "don't know specifically, but later"; or "don't know.” The participants were asked to select the most typical ways of spending their leisure time during the early elementary school years from the following categories: “games,” “studying,” “reading,” “music (singing and playing musical instruments),” and “exercise (physical play).” Participants were asked to answer the same questions on how they spent their leisure time in the middle and upper elementary school grades.

2) Sports experience

Initially, the participants were asked to choose "Yes" or "No" to the question, "Have you ever learned any physical exercise or sports? " Those who responded "yes" were asked additional questions regarding the number of events they had experienced, the age at which they started playing sports, specialized events, and athletic performance. The

participants were asked to select the number of sports events they had experienced from the options "1,” "2,” "3,” "4,” and "≥5 ". Participants were also asked to select the age at which they started learning sports from the following age groups: < 3, 3, 4, 5, 6, 7, 8, 9, 10, 11, 12, 13, 14, 15, and ≥16 years. Additionally, they were asked to select the sport in which they had the longest experience from the following categories: baseball, soccer, rugby, basketball, tennis, badminton, volleyball, track and field (short distance), track and field (long distance), swimming, table tennis, kendo, judo, and gymnastics. Regarding the competition results, the participants were asked to select from the following levels: "Prefectural Qualifier Level," "Prefectural Tournament Participant Level," "Prefectural Tournament Placement Level," and "National Tournament Participant Level. "

3) Subjective athletic ability

Subjective general athletic ability, athletic ability in each sport (volleyball, basketball, badminton, tennis, baseball, table tennis, soccer, gymnastics, short-distance race, and long-distance race), father's athletic ability, and mother's athletic ability were assessed using an 11-point scale (1 = lowest athletic ability and 11 = highest athletic ability). The participants were asked to select the age at which they became aware of their athletic ability (superiority or inferiority) from the following age groups: 4, 5, 6, 7, 8, 9, 10, 11, 12, 13, 14, 15, 16, 17, 18, and ≥19 years. Regarding evaluation by others, the participants were asked, "Have you ever been told by others that you are a good athlete? They were to choose from "often,” "sometimes,” "not often,” and "never.” The participants were asked to select their answer from "very much faster than the average person," "a little faster than the average person," "about average," "a little slower than the average person," or "very much slower than the average person" to the question "What is the average speed at which you learn a movement (technique) for the first time?”

4) Personality characteristics

For the evaluation of grit, we used the Japanese version of the Short Grit scale [27], which comprises eight items. The original version of this scale was developed in English [28], and Nishikawa et al. assessed the validity of the questionnaire translated into Japanese [27]. Responses were recorded on a five-point scale, with one indicating "not applicable" and five indicating "applicable." In this study, the total score of each item was used as the grit value for data analysis. The Cronbach's alpha coefficient for this scale was 0.7.

For the evaluation of resilience, we used the Japanese version of the Brief Resilience Scale [29], which was originally developed in English [25], and Tokuyoshi and Moriya assessed the validity of the questionnaire translated into Japanese [29]. The scale includes six items, and participants were instructed to respond using a five-point scale (1 = not applicable at all, 2 = not very applicable, 3 = undecided, 4 = somewhat applicable, and 5 = quite applicable). In this study, the total score for each question item was used as the analytical data for the resilience value. Cronbach's alpha coefficient for this scale was 0.88.

We used the Intelligence Scale as an indicator to measure growth mindset. Ten items from the Intelligence Scale were used in this study [30]. Responses were recorded on a five-point scale, with one indicating "not true" and five indicating "very true." In this study, the total score of each question item was used as analytical data for the intelligence view. Cronbach's alpha coefficient for this scale was 0.77.

3. Data analysis

Data analyses were conducted using Python (version. 3.11.1), and R (version. 4.3.2).

As part of the data pre-processing, responses that did not answer the question, such as "I don't know," were treated as missing values. In this study, a listwise exclusion method was used without completing missing values. The data used in each regression model analysis are listed respectively (Tables 1 and 2). The collected responses were converted to numbers, and dummy variables were used in the analysis when categorical data were included in the regression model. All models showed an association with subjective total athletic performance based on a nonstandardized coefficient (B).

A correlation analysis was conducted using Pearson's product-rate correlation coefficient to examine the relationship between an individual's subjective general athletic performance and their subjective athletic performance in each sport.

To identify the factors influencing gross motor skills, we conducted a linear regression analysis using an individual's subjective total motor skill, which is the total subjective athletic performance score for each discipline, as the objective variable. The lm () function of R was used to perform linear regression analysis. Nine linear regression analysis models were created, including different explanatory variables, with the subjective total exercise capacity as the objective variable. The covariates in all models were sex, age, and birth month. Japan uses a unique annual age-grouping policy for sports and education, effective between April 1 to March 31 of the following year.

Consequently, for the birth month, the numbers were adjusted such that April births began at 1 and March births ended at 12. Model 1 included three personality traits: grit, resilience, and intelligence. Model 2 included leisure-time activities in elementary schools categorized by grade level. A dummy variable was used for leisure time, with exercise as the criterion variable. Model 3 included sibling composition. A dummy variable was used, and the criterion variable was set as an only child. Model 4 included

the presence or absence of exercise experience. Model 5 incorporated variables such as the number of sports practiced over a year, age at first engagement with sports, and competition performance. A dummy variable was used for competition performance and the reference variable was the qualification level required for the prefectural tournament. Model 6 included the combined annual household income of the parents (10, 000 yen), and Model 7 included the age at which participants started walking. Model 8 included the subjective athletic performances of fathers and mothers. Model 9 included the age at which the participants became aware of the superiority of their motor skills, the frequency of being told by others that they are "athletic," and the speed at which they learned the movements (movements/techniques) they were performing for the first time. A dummy variable was used for the frequency of being told words, such as "you have good motor skills," and the criterion variable was "never been told." The speed of learning a movement (movement/technique) for the first time was also used as a dummy variable, with the criterion variable being "very slow compared with the average person." Regarding the age at which they began walking, participants were asked how old they were when they began walking. If the answer was "I don't know specifically, but about average," rather than a specific age, the answer was 12 months; if the answer was "I don't know specifically, but I was on the early side," the answer was 8 months; and if the answer was "I don't know specifically, but I was on the late side," the answer was 17 months. Responses of < 9 months were integrated with those of 8 months, and responses of > 17 months were integrated with those of 17 months. The coefficients of determination were listed as R^2 values of the adjusted coefficients of determination.

Results

The correlations between subjective general athletic ability and athletic ability in each sport and their relationships are shown in Fig 1. The analysis revealed that the general subjective athletic ability was strongly correlated with soccer, volleyball, basketball, and short-distance racing. Tables 1 and 2 present the results of the linear model analysis. The relationship between the explanatory variables used in each model and total athletic ability is illustrated in Figures 2 and 3. Model 1 demonstrated that higher scores for personality traits were associated with greater total athletic ability (F (6, 399) = 14.73, R^2 = 0.17, p < 0.001; Fig 2). Model 2 indicated that leisure activities, such as games and studying in the middle grades, along with games, reading, and music in the higher grades, were negatively correlated with total athletic ability. (F (15, 390) = 10.24, R^2 = 0.25, p < 0.001, Fig 3). In Model 3, where sibling composition was an explanatory variable, a positive association was observed between youngest siblings and total athletic ability (F (6, 399) = 6.54, R^2 = 0.08, p < 0.001; Fig 3). The results of Model 4 demonstrated a positive association between sports experience and total athletic ability (F (4, 401) = 16.69, R^2 = 0.13, p < 0.001, Fig. 3). The results of Model 5 showed that the number of sports learned for > 1 year and athletic performance were related to total athletic ability, but not the age at which the first exercise or sport was learned (F (8, 360) = 11.33, R^2 = 0.18, p < 0.001, Figs 2 and 3). Model 6, which examined the association with annual household income, revealed a positive association between total athletic ability and parental household income of the parents (F (4, 145) = 7.69, R^2 = 0.15, p < 0.001; Fig. 2). The results of Model 7 demonstrated that an earlier age at initial walking was associated with greater total athletic ability (F (4, 309) = 9.84, R^2 = 0.10, p < 0.001; Fig 2). Model 8 indicated that the subjective athletic ability of fathers 'and mothers' was associated with increased individual total athletic ability (F (5, 369) = 17.66, R^2 = 0.18, p < 0.001, Fig 2).

Model 9 revealed that individuals frequently described by others as "superior athletes" exhibited higher total athletic ability (*F* (11, 394) = 38.73, R^2 = 0.51, p < 0.001, Figs 2 and 3). Additionally, using the same model, it was observed that a faste[illegible]tial learning speed of a movement (technique or skill) was positively associated with greater total athletic ability.

Fig 1 Correlation between subjective general athletic ability and subjective athletic ability in each sport. For dots, lines, and box plots, blue indicates males and red indicates females, respectively. The numbers in the upper right-hand corner indicate correlation coefficients. ***: $p < 0.001$, **: $p < 0.01$

Fig 2 Relationship between personality traits and subjective total athletic ability. The horizontal axis shows each explanatory variable, and the vertical axis shows total athletic ability. The dots in the figure represent individual observations, with red and blue indicating females and males, respectively. The straight lines indicate regression lines based on the least-squares method, and the gray bands around the lines indicate 95% confidence intervals of the regression lines.

Fig 3 Relationship between leisure activities and subjective total athletic ability. The horizontal axis shows each category of explanatory variables, and the vertical axis shows total athletic ability. The dots in the figure represent individual observations, and each color represents a different leisure activity.

Table 1. Results of multiple regression analysis in subjective overall motor performance (Models 1 – 4)

	Model 1		Model 2		Model 3		Model4	
Variables	B	95% CI	B	95% CI	B	95% CI	B	95% CI
(Intercept)	-12.903	[-46.215, 20.410]	44.231**	[13.565, 74.896]	15.626	[-18.512, 49.763]	14.993	[-17.506, 47.491]
Sex	7.638***	[4.504, 10.772]	5.966***	[2.823, 9.110]	8.197***	[4.894, 11.501]	7.206***	[4.021, 10.391]
Age	1.544+	[-0.153, 3.241]	0.841	[-0.787, 2.470]	1.545+	[-0.259, 3.349]	1.159	[-0.578, 2.896]
Birth Month	0.135	[-0.329, 0.599]	0.039	[-0.410, 0.487]	0.090	[-0.399, 0.580]	0.017	[-0.456, 0.490]
**Personality**								
Grit	0.404**	[0.120, 0.687]						
Resilience	0.586***	[0.340, 0.832]						
Inteligence	0.409**	[0.160, 0.658]						
**Leisure Time**								
Lower Grade								
Game			-0.221	[-5.268, 4.825]				
Reading			-5.934+	[-11.984, 0.117]				
Music			-4.314	[-14.234, 5.606]				
Study			1.840	[-7.975, 11.655]				
Middle Grade								
Game			-8.297***	[-13.194, -3.399]				
Reading			-5.972+	[-12.628, 0.684]				
Music			-3.989	[-14.290, 6.311]				
Study			-15.317**	[-24.993, -5.642]				
Higher Grade								

Game			-7.877***	[-11.987, -3.767]				
Reading			-10.659***	[-16.790, -4.529]				
Music			-11.770**	[-20.035, -3.505]				
Study			-4.645	[-10.616, 1.326]				
**Birth order**								
Only Child					3.932	[-3.595, 11.458]		
Oldest Child					5.409	[-2.815, 13.632]		
Middle Child					8.229*	[0.689, 15.768]		
Youngest Child								
**Sport History**							16.109***	[10.641, 21.577]

	Model 1	Model 2	Model 3	Model4
Num.Obs.	406	406	406	406
R2	0.181	0.283	0.090	0.143
R2 Adj.	0.169	0.255	0.076	0.134

***: p < 0.001, **: p < 0.01, *: p < 0.05, +: p < 0.1

Table 2. Results of multiple regression analysis in subjective overall motor performance (Models 5 – 9)

	Model 5		Model 6		Model 7		Model 8		Model 9	
Variables	B	95% CI	B	95% CI	B	95% CI	B	95% CI	B	95% CI
(Intercept)	13.937	[-17.824, 45.698]	-22.322	[-77.827, 33.184]	53.039**	[12.811, 93.266]	-4.408	[-38.350, 29.533]	8.773	[-16.553, 34.099]
Sex	3.579*	[0.366, 6.793]	9.777***	[4.140, 15.414]	8.746***	[4.949, 12.543]	9.957***	[6.717, 13.196]	6.834***	[4.380, 9.288]
Age	1.363	[-0.304, 3.031]	2.451	[-0.505, 5.408]	0.740	[-1.334, 2.814]	1.685+	[-0.080, 3.450]	0.949	[-0.367, 2.265]
Birth Month	0.078	[-0.380, 0.536]	0.799+	[-0.001, 1.599]	-0.027	[-0.580, 0.527]	0.104	[-0.381, 0.590]	-0.173	[-0.533, 0.186]
Sport Kind	3.164***	[1.733, 4.595]								
**Competition**	0.451	[-0.180, 1.082]								
Prefectural Qualifier Level	8.925***	[5.187, 12.663]								
Prefectural Tournament Level	13.326***	[8.321, 18.332]								
Prefectural Award Level	8.452**	[2.555, 14.349]								
National Tournament Level										
**Income**			0.018**	[0.007, 0.028]						
Walking Age					-1.310***	[-2.024, -0.595]				

**Parents' Ability**										
Father Ability							1.764***	[0.962, 2.566]		
Mother Ability							1.616***	[0.920, 2.312]		
**Others**										
Recognition Age									0.123	[-0.279, 0.525]
Compliment										
Not often said									9.439***	[5.316, 13.561]
Sometimes said									17.317***	[13.361, 21.274]
Often said									25.976***	[21.338, 30.613]
Motor Learning Speed										
Little Slow									7.138**	[1.784, 12.491]
Average									10.847***	[5.318, 16.375]
Little Fast									13.376***	[7.704, 19.048]
Too Fast									15.023***	[7.935, 22.110]

	Model 5	Model 6	Model 7	Model 8	Model 9
Num.Obs.	369	150	314	375	406
R2	0.201	0.175	0.113	0.193	0.519

R2 Adj.	0.183	0.152	0.102	0.182	0.506

***: $p < 0.001$, **: $p < 0.01$, *: $p < 0.05$, +: $p < 0.1$

Tables 1 and 2 present the models used in multiple regression analyses. Sex, age, and birth month were used as covariates.

B: Standardizing Coefficient, CI: Confidence Interval

Discussion

Awareness of proficient motor skills may encourage individuals to engage in physical activity, which could be crucial to their athletic careers, particularly during their youth. However, the concept of motor skills remains ambiguous and lacks a clear definiti[illegible] Motor skills are often referred to as an individual's overall athletic ability; however, this term is subjective and may encompass various aspects of physical competence. In this study, we aimed to conduct a questionnaire survey among college students to investigate the relationship between their subjective general athletic ability (commonly referred to as motor skills) and their subjective athletic ability in various sports, personality traits, sibling composition, socio-economic status, and athletic performance. Subjective general athletic ability was highly correlated with subjective athletic ability in short-distance races such as volleyball, basketball, and soccer. Furthermore, various elements such as personality traits, leisure-time activities, and sibling composition were associated with total athletic ability.

We found that an individual's exercise experience influenced their total athletic ability. According to a study that investigated high and low motor competence, exercise habits, and health-related indicators among college students [31], students in the low motor competence group were more likely to have no experience with exercise club activities, whereas those in the high motor competence group were more likely to have exercise habits. Engaging in sports outside of physical education classes during regular school activities probably leads to improvements in physical fitness and athletic abilities, resulting in an improvement in total athletic ability. The various sports practiced for more

than a year and the level of athletic performance was linked to total athletic ability rather than the age at which they first began participating in exercise or sports. A study [32] that investigated the effects of youth sports life careers on the frequency of sports in adulthood showed that adults who had played multiple sports during their elementary, junior high, and high school years played sports more frequently than those who had played a single sport. Therefore, the practice of multiple sports might have led to an increased frequency of sports in adulthood, and total athletic ability improved as the participants experienced various physical activities and acquired sports skills.

Previous studies on short-distance racing have reported that children with better short-distance racing records have a higher sense of motor competence [33]. In short-distance racing, individuals can easily compare themselves with others in terms of their times and rankings. This could lead to a clearer evaluation of athletic ability. Consequently, short-distance racing is a sport in which athletic ability can be judged objectively based on the perceived difference in ability compared with others. A study of junior high school students [34] showed that despite improvements in short-distance racing records as they grew older, their rating scores for "good at short distance race," which indicates perceptions of physical competence, dropped significantly. Compared with other sports, it is relatively difficult to feel a genuine sense of improvement in skills, and easy to feel limited in improving records. This study suggests that individuals with high subjective abilities in short-distance races also exhibit high subjective abilities in other sports. However, compared with other sports, short-distance racing reflects an individual’s physical ability rather than skill and experience, and further studies are required to

determine the effects of short-distance racing ability on other sports.

A study [35] examined the overall ball gaming ability and motor skills of college students and reported that those who were good at ball games rated their motor skills as good. Subjective athletic abilities in soccer, volleyball, and basketball were highly correlated with subjective general athletic ability. Individuals with high athletic ability are likely to have a sense of athletic competence in popular sports, especially considering the intense competition within teams during club activities. Studies examining various sports and the relative age effect [19,21] have reported a strong effect in major sports but not minor sports. Relative age effects are strongly correlated with motor competence [36], and the sense of motor competence may be enhanced by being good at major sports, leading to improved ability in various sports (subjective general athletic ability).

The relationship between subjective general athletic ability and exercise category suggests that high motivation to improve motor skills is linked to high subjective athletic ability. Personality traits such as grit and resilience are thought to be the ability to sustain effort when placed in difficult situations in the process of achieving long-term goals [37]. High competence in these personality traits is considered the ability to persevere in acquiring superior skills. In addition, the personality trait of persistence is involved in maintaining a high level of motivation for improvement, which is necessary to consistently achieve goals. Furthermore, the view of intelligence examined in this study reflects a mindset that believes that talent can be changed through hard work [30]. We speculate that the influence of views of intelligence on total athletic ability observed in this study may have resulted in higher total athletic ability as a reflection of the ability to

continue working hard to acquire advanced skills.

This study revealed the importance of leisure time in elementary school on subjective athletic ability. Compared with physical activity, games, and studying in the middle grades, leisure activities other than exercise (games, reading, and music) in the higher grades negatively affected total athletic ability. A study of lifestyle habits in various countries [38] reported that spending > 2 h daily playing video games decreased the habit of moderate to vigorous physical activity. Sedentary lifestyle habits such as television, video, and video games in elementary school children, body mass index, and abdominal circumference were significantly reduced in the intervention group that reduced these habits [39]. Therefore, leisure time spent sedentarily, such as playing games or reading books, leads to decreased total athletic ability. Physical activity during childhood may be significant in the development of motor-skill proficiency in adulthood.

There was a positive association between being the youngest siblings and subjective athletic ability. Previous studies with young children have reported that being with other children involves more physical activity than being alone among 3–6-year-olds [40] and that living with other children of the same age or older contributes to improved overall athletic ability [41]. Similarly, it has been shown that younger siblings (younger brothers/sisters) exhibit better athletic ability than only children [12]. Studies on preschool siblings have also reported that when older children learn to crawl, walk, and climb, younger children frequently imitate their actions [42[illegible]hese studies suggest that the youngest children who grew up in an environment in which siblings of the same or older age were always present at home were more physically active and had more

opportunities to imitate their older siblings' movements, leading to a relative increase in their total athletic abilities.

Considering that sibling composition is associated with total athletic ability, family involvement is a significant factor in the development of motor skills. This study found that the subjective athletic abilities of the fathers and mothers were associated with their total athletic abilities. Genetic and environmental factors are the two main factors behind the association between parental and total athletic abilities. Studies examining the heritability of athletic performance have reported 56% heritability of maximal oxygen uptake [43] and 52% heritability of muscle strength and power [44]. This indicates that genetic factors explain over 50% of individual differences in athletic performance. Based on these studies, parents' athletic ability may have had a genetic influence on their children's athletic ability. Furthermore, Xu et al. [45] reported a positive correlation between parents' and children's physical activity levels and the influence of parents' perceptions of the importance of physical activity on children's physical activity. Parents with high athletic abilities may have a better understanding of the importance of physical activity for their children, potentially impacting their children's total athletic ability.

The positive association between total athletic ability and household income suggests that household economic status positively affected total athletic ability. Previous studies have shown that higher socioeconomic status is associated with higher physical fitness levels [15] and that families with higher annual household incomes are more likely to engage their children in sports activities [46]. Therefore, the more economically affluent the family, the greater the opportunities for sports activities, the higher the level of

physical fitness, and the greater the total athletic ability.

The relationship between the age at which they started walking and their total athletic ability suggests that the development of physical function during childhood influences athletic ability later. A study by Oikawa [47] that investigated the effects of infant motor development, such as walking and holding the head, on athletic ability in early childhood showed that the earlier the age at which walking begins, the more positive the effect on boys' running and throwing abilities. Age at the initiation of walking is also associated with grip strength in middle age [48] and with muscle and cardiovascular endurance in mature age [49]. Consistent with these previous studies, an earlier age at walking initiation may improve the physical ability of college students.

Furthermore, the evaluation of good motor skills by others was associated with a higher total athletic ability. The results obtained in this study indicate the importance of evaluations by other researchers. Since positive feedback from others enhances intrinsic motivation [50], positive feedback on athletic ability probably triggers increased intrinsic motivation for exercise and improves total athletic ability. We also observed that the faster the speed of learning a movement (action/technique) for the first time, the higher the total athletic ability. Physical education and sports activities in schools and clubs often occur in groups. The experience of being able to learn new skills faster than others might contribute to the development of a positive attitude toward sports and improvement in total athletic ability.

This study has some limitations. There may be some bias in the parents' income and sample because this study was conducted only with students at a particular college.

Moreover, parents' athletic abilities affect their children's athletic abilities; however, this influence cannot be completely eliminated. In addition, this study was conducted with participants who subjectively evaluated their athletic abilities. In the future, more detailed studies will be possible by combining objective evaluation indices. Furthermore, as this study used a cross-sectional design to obtain data, we could not interpret the causal relationship between the factors associated with total athletic ability found in this st[illegible]y. A longitudinal study is needed to determine changes in total athletic ability to identify factors that may enhance motor skills. This study examined the factors associated with motor skills by employing various types of linear models; however, it did not ascertain the extent to which each factor contributed to total athletic ability. Therefore, additional studies are required to confirm these findings.

Conclusion

We aimed to identify the multiple factors associated with subjective total athletic ability (commonly referred to as motor skills) among undergraduate students. Our results indicated that various factors were associated with subjective total athletic ability, including personality traits such as grit, resilience, and a sense of intelligence; how they spend their leisure time; sibling composition; previous exercise experience; parents' athletic ability; household income; how often they are called "athletic" by others; and age when they started walking. Therefore, it is necessary to clarify how these factors can determine good or bad motor skills from a comprehensive perspective. Based on the relationships between motor skills and the associated factors identified in this study,

multiple factors may be intricately associated with the development of a sense of motor skills.

Acknowledgments

We would like to thank all the participants of the study. We are grateful for this comment.

References

1. Warburton DE, Nicol CW, Bredin SS. Health benefits of physical activity: the evidence. CMAJ. 2006;174: 801-809. doi: 10.1503/cmaj.051351.
2. Storm RK, Eske M. Dual careers and academic achievements: does elite sport make a difference? Sport Educ Soc. 2022;27: 747-760. doi: 10.1080/13573322.2021.1919070.
3. Nakayama A, Matsuzaka A, Yoshino S. Perceived competence in sport and exercise, physical fitness, and motor skills among primary school children. Ibaraki Univ Stud Teach Strategy. 2012;31: 255-262. (in Japanese).
4. Ueno K, Seki K. Relationship between physical strength and perceived physical competence is influenced by sex differences and developmental tendency. Sanin J Phys Educ. 2011;26: 13-18. (in Japanese).
5. Okazawa Y, Kita M, Suwa Y. Factorial structure of physical competence and its developmental tendency and sex difference. Jpn J Sport Educ Stud. 1996;16: 145-155.

(in Japanese). doi: 10.7219/jjses.16.145.
6. Takeda M. The relationship between “physical fitness” and “physical competence” of elementary school pupils. J Morioka Univ. 2005;22: 41-47. (in Japanese).
7. Takeda M. The relationship between “physical fitness” and “physical competence” of elementary school pupils (II). J Morioka Univ. 2006;23: 67-74. (in Japanese).
8. Ono Y, Kaji M. Development of the physical competence scale for elementary school students in Japan. Int J Sport Health Sci. 2020;18: 122-133. doi: 10.5432/ijshs.201906.
9. Strano DA, Petrocelli JV. A preliminary examination of the role of Inferiroty feelings in the academic achievement of college students. J Individ Psychol. 2005;61: 80-89.
10. Yamashita S, Hirano T, Asakawa M. The infantile life and environment factor in connection with growth of athletic ability. Bulletin of Okazaki women's university. 2013; 47, 25-32. (in Japanese).
11. Krombholz H. Physical performance in relation to age, sex, birth order, social class, and sports activities of preschool children. Percept Mot Skills. 2006;102: 477-484. doi: 10.2466/pms.102.2.477-484.
12. Berger SE, Nuzzo K. Older siblings influence younger siblings' motor development. Infant Child Dev. 2008;17: 607–615. doi: 10.1002/icd.571.
13. Casher BB. Relationship between birth order and participation in dangerous sports. Res Q. 1977;48: 33-40. doi: 10.1080/10671315.1977.10762146.
14. Shimokubo T. The effect of socio-economic status of family of origin on exercise habits in adulthood: analysis of direct and indirect effects based on individual socio-economic status. Japan J. Phys. Educ. Hlth. Sports Sci. 2023;68: 87-102. (in Japanese).

15. Wolfe AM, Lee JA, Laurson KR. Socioeconomic status and physical fitness in youth: findings from the NHANES National Youth Fitness Survey. J Sports Sci. 2020;38: 534-541. doi: 10.1080/02640414.2020.1713688.

16. Kimura M, Ogata M. Effect of wrestling on elementary school children’s physical competence and exercise habits. Jpn J Coaching Stud. 2021;35: 75-89. (in Japanese).

17. Goto Y, Kakehata G, Tagami K. Development of teaching running form to improve physical competence in elementary school children. Sasakawa Sports Res Grants. 2018: 314-320. (in Japanese).

18. Fukami E, Nobushima S, Suzuki K, Shimazaki T. An experimental study of acquiring an effective sprinting movement in upper elementary grade PE classes – focusing on enhancing students’ sports competence. Sports Sci Res. 2017;14: 1-12. (in Japanese).

19. Dudink, A. Birth date and sporting success. Nature. 1994; 368, 592. doi: 10.1038/368592a0

20. Borg MG, Falzon JM. Birth date and sex effects on the scholastic attainment of primary schoolchildren: A cross-sectional study. British Educational Res J. 1995;21: 61-74. doi: 10.1080/0141192950210105.

21. Nakata H, Sakamoto K. Relative age effects in Japanese athletes. J Phys Fit Sports Med. 2014;3: 467-476. doi: 10.7600/jpfsm.3.467.

22. Cormier DL, Ferguson LJ, Gyurcsik NC, Briere JL, Dunn JG, Kowalski KC. Grit in sport: A scoping review. Int Rev Sport Exer Psychol. 2021: 1-38. doi: 10.1080/1750984X.2021.1934887.

23. Duckworth AL, Peterson C, Matthews MD, Kelly DR. Grit: perseverance and passion

for long-term goals. J Pers Soc Psychol. 2007;92: 1087–1101. doi: 10.1037/0022-3514.92.6.1087.

24. Larkin P, O'Connor D, Williams AM. Does grit influence sport-specific engagement and perceptual-cognitive expertise in elite youth soccer? J Appl Sport Psychol. 2016;28: 129-138. doi: 10.1080/10413200.2015.1085922.
25. Smith BW, Dalen J, Wiggins K, Tooley E, Christopher P, Bernard J. The brief resilience scale: assessing the ability to bounce back. Int J Behav Med. 2008;15: 194-200. doi: 10.1080/10705500802222972.
26. Dweck CS, Yeager DS. Mindsets: A view from two eras. Perspect Psychol Sci. 2019;14: 481-496. doi: 10.1177/1745691618804166.
27. Nishikawa K, Okugami S, Amemiya T. Development of the Japanese short grit scale （grit-S）. Jpn J Pers. 2015;24: 167-169. (in Japanese). doi: 10.2132/personality.24.167.
28. Duckworth AL, Quinn PD. Development and validation of the Short Grit Scale (GRIT–S). J Pers Assess. 2009;91: 166–174. doi: 10.1080/00223890802634290.
29. Tokuyoshi Y, Moritani M. Development and validation of the Brief Resilience Scale. Japanese version (BRS-J). The proceedings of the 79th Annual Convention of the Japanese Psychological Association; 354. (in Japanese); 2015.
30. Watanabe K. Students' growth mindset development in college (I) Osaka University of Economics Departmental Bulletin Paper. 2017;68: 1-24. (in Japanese).
31. Kobayashi K, Hiiragi Y. Survey of feelings of physical competence and exercise habits and health-related indicators journal of physical therapy science. 2018;33: 55-

58. (in Japanese).

32. Hayashida T, Shimizu N. The influence of sports life career during adolescence on sports participation in adulthood: as mediation to awareness and attitude toward sports. Japan J. Phys. Educ. Hlth. Sports Sci. 2021;66: 715-736. (in Japanese).
33. Dei Y. Motor ability in children who are weak in exercise: the relation between exercise competence and physical strength. Meiji Gakuin University Departmental Bulletin Paper. 2014;24: 47-62. (in Japanese).
34. Otsuka M. Short-distance running classes in terms of attitude measurement-The Influence of School Stage, Gender, and Favorability on Classroom Evaluation [Taidosokutei ni miru tankyorisou no zyugyou – gakkou dankai, seibetu, koukando no tigai ga zyugyouhyouka ni ataeru eikyou]. Pedagog Stud Phys Educ [Taiiku-Ka Kyouiku-Gaku Kenkyu]. 2013;29: 49-62. (in Japanese).
35. Aoyama S. Study on the university students' consciousness of their motor activity— on the self-estimation of the so-called motor skill ー. The proceedings of the department of physical education, college of general education; 10. University of Tokyo; 1976. pp. 35-48. (in Japanese).
36. Kawata Y. A study on relative age effects on effort to foster participation in physical activities among elementary school children. Sasakawa Sports Res Grants. 2014: 307-314. (in Japanese).
37. Biggs AT, Seech TR, Johnston SL, Russell DW. Psychological endurance: how grit, resilience, and related factors contribute to sustained effort despite adversity. J Gen Psychol. 2023: 1-43. doi: 10.1080/00221309.2023.2253955.

38. Melkevik O, Torsheim T, Iannotti RJ, Wold B. Is spending time in screen-based sedentary behaviors associated with less physical activity: a cross national investigation. Int J Behav Nutr Phys Act. 2010;7: 46. doi: 10.1186/1479-5868-7-46.
39. Robinson TN. Reducing children's television viewing to prevent obesity: a randomized controlled trial. JAMA. 1999;282: 1561-1567. doi: 10.1001/jama.282.16.1561.
40. Barkley JE, Salvy SJ, Sanders GJ, Dey S, Von Carlowitz KP, Williamson ML. Peer influence and physical activity behavior in young children: an experimental study. J Phys Act Health. 2014;11: 404-409. doi: 10.1123/jpah.2011-0376.
41. Barnett LM, Hnatiuk JA, Salmon J, Hesketh KD. Modifiable factors which predict children's gross motor competence: A prospective cohort study. Int J Behav Nutr Phys Act. 2019;16: 129. doi: 10.1186/s12966-019-0888-0.
42. Erbaugh SJ, Clifton MA. Sibling relationships of preschool-aged children in gross motor environments. Res Q Exer Sport. 1984;55: 323-331. doi: 10.1080/02701367.1984.10608410.
43. Miyamoto-Mikami E, Zempo H, Fuku N, Kikuchi N, Miyachi M, Murakami H. Heritability estimates of endurance-related phenotypes: A systematic review and meta-analysis. Scand J Med Sci Sports. 2018;28: 834-845. doi: 10.1111/sms.12958.
44. Zempo H, Miyamoto-Mikami E, Kikuchi N, Fuku N, Miyachi M, Murakami H. Heritability estimates of muscle strength-related phenotypes: A systematic review and meta-analysis. Scand J Med Sci Sports. 2017;27: 1537-1546. doi: 10.1111/sms.12804.
45. Xu H, Wen LM, Rissel C. Associations of parental influences with physical activity

and screen time among young children: a systematic review. J Obes. 2015;2015: 546925. doi: 10.1155/2015/546925.

46. Benesse Education Research and Development Institute. First survey of school education activities; 2009 (Databook). [Cited 2024 April 17]. Available from: https://berd.benesse.jp/shotouchutou/research/detail1.php?id=3265. (in Japanese) [Daiikkai gakkou kyouikukatsudou ni kansuru chousa 2009 (de-ta bukku)].
47. Oikawa N. Associations of Birth Size and Motor Development in Infancy with Motor Ability in 4-6-year-olds. J Child Health. 2018;77: 142-148. (in Japanese).
48. Kuh D, Hardy R, Butterworth S, Okell L, Wadsworth M, Cooper C, et al. Developmental origins of midlife grip strength: findings from a birth cohort study. J Gerontol A Biol Sci Med Sci. 2006;61: 702-706. doi: 10.1093/gerona/61.7.702.
49. Ridgway CL, Ong KK, Tammelin T, Sharp SJ, Ekelund U, Jarvelin MR. Birth size, infant weight gain, and motor development influence adult physical performance. Med Sci Sports Exerc. 2009;41: 1212-1221. doi: 10.1249/MSS.0b013e31819794ab.
50. Matsui K. Students' Intrinsic Motivation in High School Athletic Clubs: the role of students' perceptions of the student-coach relationship and coaches' feedback. Jpn J Sport Psychol. 2014;41: 51-63. doi: 10.4146/jjspopsy.2013-1301.

Supporting information

Figure 1

Click here to access/download;Figure;Fig. 1.TIF

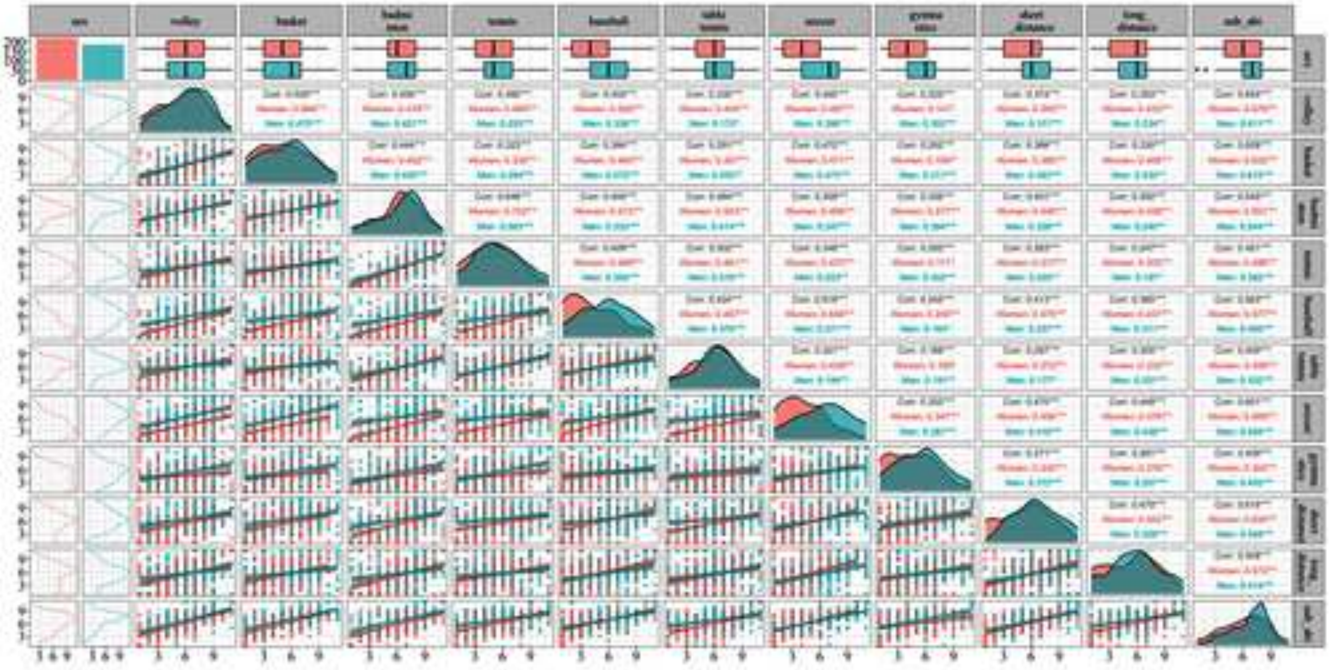

Figure 2

Click here to access/download;Figure;Fig. 2.TIF

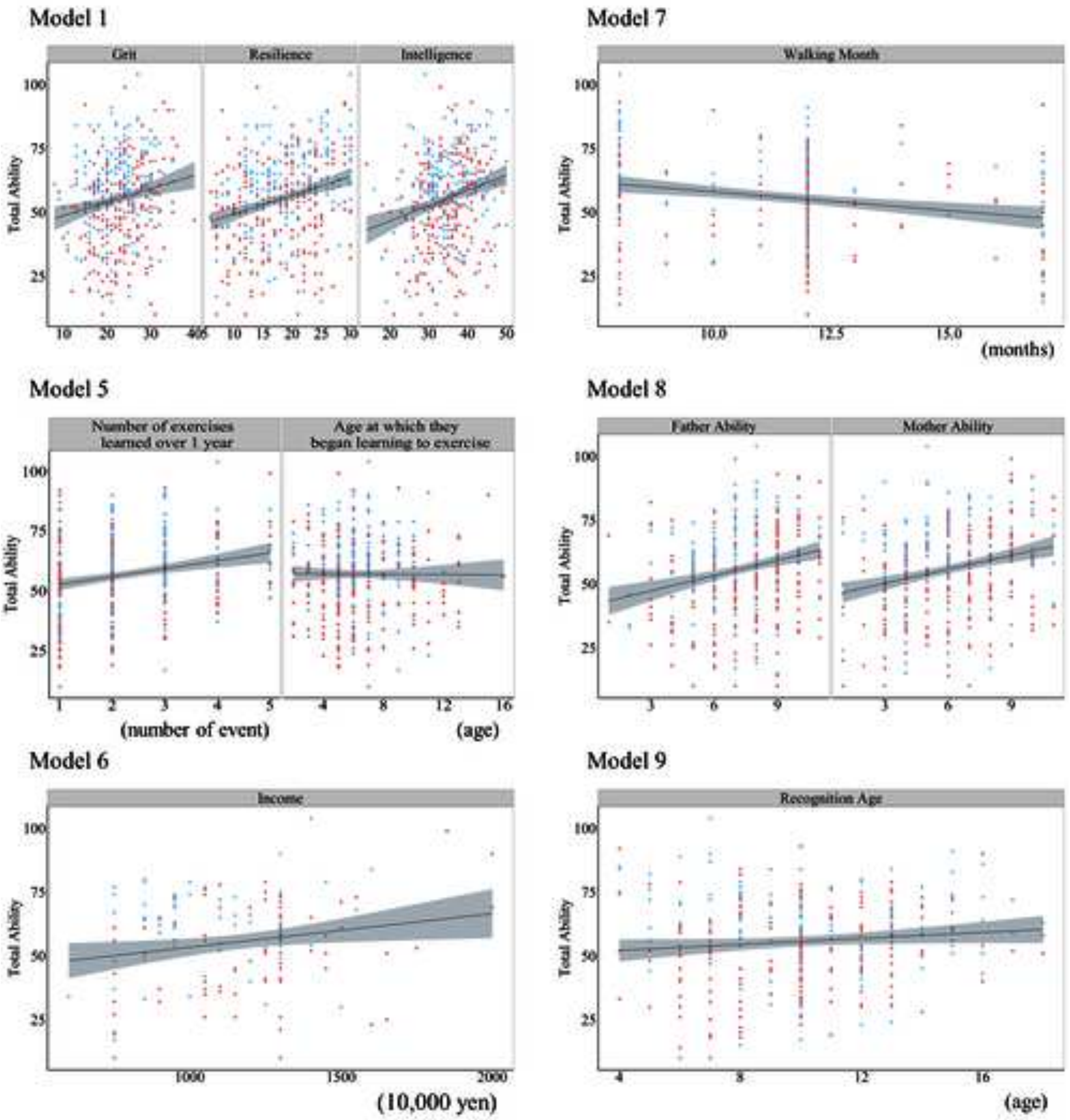

Figure 3

Click here to access/download;Figure;Fig. 3.TIF

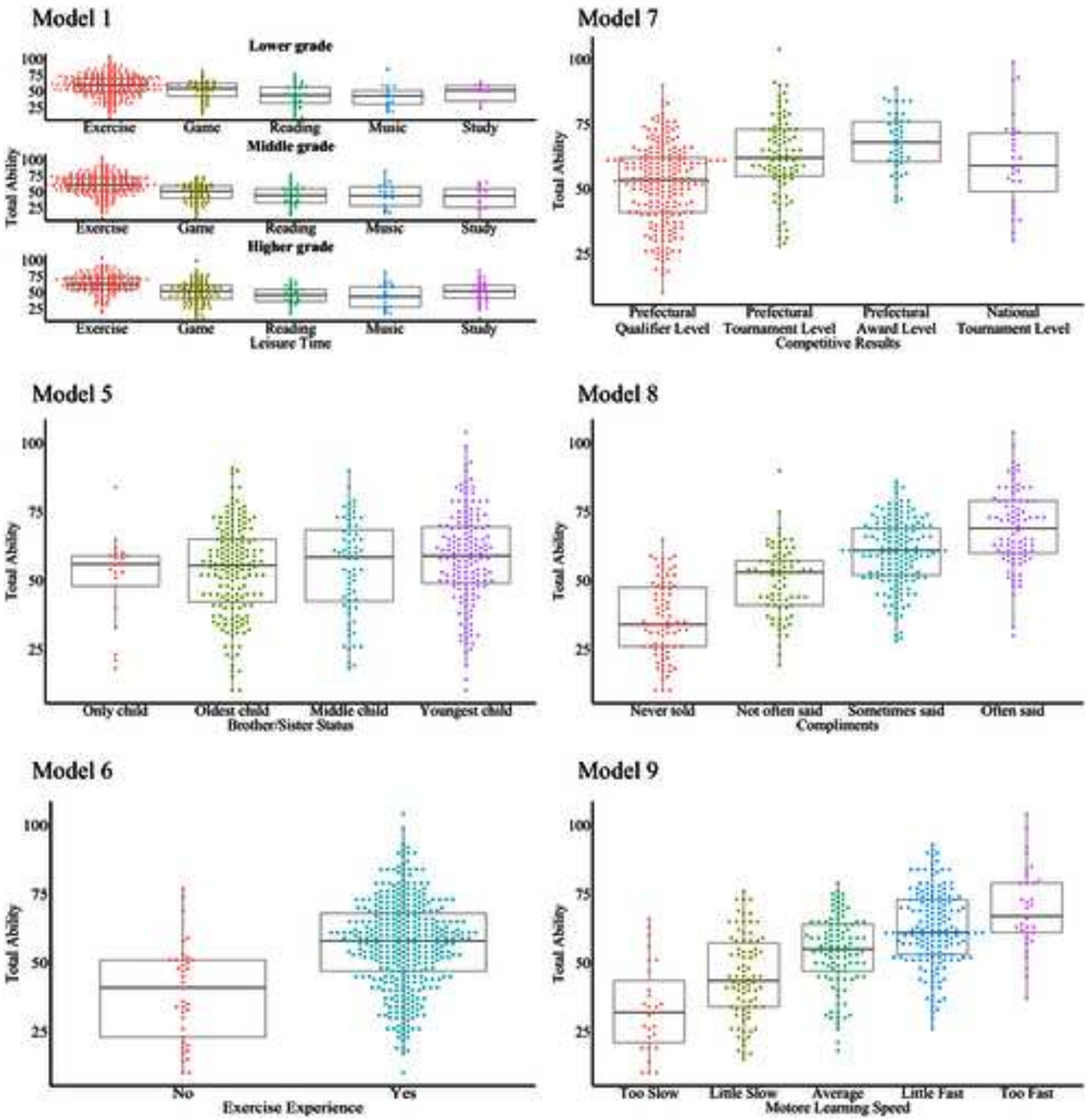

Supporting Information_alldata

Click here to access/download
Supporting Information
df_all_2.xlsx

Click here to access/download
Supporting Information
Motor_Skill_PlosOne_submit.R
