# Supplementary material for: Determinants of subjective total athletic ability
Source: PLoS One. 2025 May 28;20(5):e0324044. doi: 10.1371/journal.pone.0324044 (PMC12118886; doi:10.1371/journal.pone.0324044)
Supplement: Attachment — Submitted filename: Response to Reviewers PONE-D-24-20019.docx [file pone.0324044.s006.docx]

Reviewer #1: BRIEF SUMMARY

This paper presents the findings about the determinants of subjective (perceived) athletic ability. The study aimed to conduct a questionnaire survey among college students to determine the relationship between their subjective general athletic ability (commonly referred to as motor skills) and their subjective athletic ability in various sports, personality traits, sibling composition, socio-economic status, and athletic performance.

The current manuscript does not replicate articles in English, and the few close articles on this topic can only be found in Japanese. The manuscript displays some degree of originality, making it potentially beneficial for the English-speaking community.

The weakness of the paper is that it is a descriptive cross-sectional study.

Thank you very much for your time to review this manuscript for improvement. We have revised the manuscript based on your comments, and we believe these modifications have all been thoroughly addressed. We have included our responses to your comments below. All correlations are highlighted in red in our manuscript.

GENERAL COMMENTS FOR AUTHORS

ABSTRACT

In research, researchers often observe a group of participants over a long period of time, called a cohort. It is therefore inappropriate to use the term “cohort” in a cross -sectional study.

Thanks for your valuable comments. We changed the sentence to read as (L30-L32)

A sample of 406 undergraduate students completed a questionnaire to evaluate their perceived athletic prowess in various sports, as well as assessments of their personality traits, family background, and sports performance.

BACKGROUND OF THE STUDY

The literature in Japanese reflects research and approaches that go back more than ten years, but today's understanding may already be much more refined and profound. It would therefore be more useful to focus on this area, with an emphasis on recent publications.

A real rationale for the study is presented but no hypotheses are offered. As a result, it is not clear why the study should be conducted, how it is built on previous research (and overcomes some of the problems of previous work), what results are expected.

My principal recommendation is that the introduction should clearly state the research hypothesis(-es).

Thanks for your valuable comments.

We have revised the references to include more recent literature where possible, ensuring that the manuscript reflects the most current understanding in this area. However, we have retained certain references older than ten years as they represent foundational or seminal works crucial to the context and framework of this study. These older references are still widely cited and relevant in the field.

Reference number 3: Ueta K, Miyao N, Otomo S. Impacts of short-term exercises interventions on grip strength,ball throwing for distance and physical competence in school children. Japanese Journal of Sport Education Studies. 2020;39 (2): 1-11. (in Japanese).

Reference number 4: Matsui T, Kubota H, Tokuyama H, Kimura M. Research on physical fitness, athletic ability and physical competence in University students. Bull. Tokyo Gakugei Univ. Division of Arts and Sports Sciences. 2019;71: 131-136. (in Japanese).

Reference number 11: Miyata H, Tsujikawa H, Kumakura T, Suzuki K. Influence of Preschooler’s Lifestyle and Family Support on Physical Fitness Development. Bulletin of Tokyo University and Graduate School of Social Welfare. 2021; 11, 13-23. (in Japanese).

Reference number 34: Oishi K, Ube H. Relationship between new physical fitness test and physical competence of junior high schooler students in remote area. Journal of Children's Physical Health 1. 2024; 51-58. (in Japanese).

In line with these updates, we have made the following adjustments (L85-L94).:

Furthermore, based on studies conducted among university students, it was found that women with higher perceptions of motor competence also exhibited greater physical fitness. However, no significant difference in physical fitness was observed between men with high and low perceptions of motor competence. The authors suggest that this may be due to the influence of sports club experience and specialized athletic skills, which are not assessed by general physical fitness tests. This test focuses on basic motor abilities such as jumping, running, and throwing [4]. This indicates that differences in sports experience may significantly influence subjective athletic ability, or that individuals can estimate their physical abilities with relative accuracy.

Additionally, we added the hypothesis at the end of the introduction and the part now read as (L113-L117).

We hypothesized that subjective athletic ability among college students is influenced by personality traits, family environment (e.g., sibling composition), and prior athletic performance. For example, students with a history of participation in competitive sports may report higher subjective athletic ability compared to those without such experience.

RESEARCH METHODS

The section is missing some important information.

Representativeness – statistical power (sample size calculation), should be presented.

Thank you for your suggestion. We have added the satirical power in our manuscript (L209-L213)

We calculated the required sample size using G*Power (version. 3.1) with the following parameters: a relatively small effect size (f² = 0.04), α = 0.05, power = 0.80, and 8 predictors (the maximum number of variables in our model). The analysis indicated that a total sample size of 384 was required. Our actual sample size (N = 406) exceeded this requirement, confirming that it was sufficient to detect the hypothesized effects.

Please describe inclusion and exclusion criteria.

We would like to clarify the selection criteria for our study participants:

Inclusion criteria:

・Participants who provided informed consent for the study

・Participants who responded to the sex/gender question

Exclusion criteria:

・Those who did not consent to participate in the study

・Those who did not provide a clear response to the sex/gender question or left it blank

Sex/gender data was essential as it was included as a covariate in our analysis, necessitating clear responses from all participants included in the final analysis. Our analytical sample was thus limited to participants who both provided consent and had clear sex/gender data available.

The above explanation is provided in materials and methods section of the text.

Quantitative indices of instruments validity and reliability must be provided (not only Cronbach alphas but also Mc Donalds omegas).

Thank you for your valuable suggestions. We have added the McDonald's omega coefficients in our manuscript (L234-L237):

Grit: For reliability, Cronbach's α was .70 and McDonald's ω total was .76.

Resilience: For reliability, Cronbach's α was .88 and McDonald's ω total was .89.

Intelligence Scale: For reliability, Cronbach's α was .77 and McDonald's ω total was .77.

Please indicate that study variables were checked for normality assumptions (for instance, using calculation of Skewness and Kurtosis for all study variables).

We appreciate your comments and suggestion. We examined the normality of all study variables through both visual inspection (histograms with density plots) and numerical indices (skewness and kurtosis). As shown in Supplemental 4, the distribution of all numeric variables demonstrated levels of skewness and kurtosis.

Have some assumptions for linear regression analysis been tested and met (For instance, independence (The Durbin-Watson Statistic), homoscedasticity (The Breusch-Pagan test))?

Thank you very much for your suggestions. We tested the key assumptions for linear regression analysis across all models. The Durbin-Watson test values ranged from 1.82 to 2.08 (*p*s > 0.5), indicating that the independence assumption was met. Although Model 8 showed a marginal value (DW = 1.82), it still did not exceed the conventional significance level of .05. Similarly, the Breusch-Pagan tests were not significant across all models (.18 ≤ *p*s ≤ .83), confirming that the homoscedasticity assumption was satisfied. These results indicated that our data met the basic assumptions for linear regression analysis. We have incorporated this explanation into our manuscript. (L286-L293)

Table 3 presents the diagnostic test results for the assumptions of each model. The Durbin-Watson test was conducted to assess autocorrelation in the residuals, with values ranging from 1.82 to 2.08. Most models showed no autocorrelation, except for Model 7, which indicated marginal autocorrelation. Additionally, the Breusch-Pagan test was performed to evaluate homoscedasticity, and all models demonstrated non-significant results (p > .05), confirming this assumption. For Model 7, the Durbin-Watson test was repeated 10 times, with all p-values exceeding .05, supporting the independence assumption.

**Table 3. Model assumptions and diagnostic test results**

| **Model** | **Durbin-Watson (DW)** | **Autocorrelation** | **Breusch-Pagan p-value** | **Homoscedasticity** | **Additional Notes** |
| --- | --- | --- | --- | --- | --- |
| Model 1 | 1.9 | No autocorrelation | 0.41 | Confirmed |  |
| Model 2 | 1.9 | No autocorrelation | 0.49 | Confirmed |  |
| Model 3 | 1.97 | No autocorrelation | 0.83 | Confirmed |  |
| Model 4 | 2.02 | No autocorrelation | 0.7 | Confirmed |  |
| Model 5 | 2.08 | No autocorrelation | 0.18 | Confirmed |  |
| Model 6 | 2.03 | No autocorrelation | 0.54 | Confirmed |  |
| Model 7 | 1.82 | Marginally autocorrelation | 0.68 | Confirmed | Repeated Durbin-Watson test 10 times; all p > .05 |
| Model 8 | 2.02 | No autocorrelation | 0.25 | Confirmed |  |

Why these and not others statistical methods (why the lm () function of R was used to perform linear regression analysis?), as well as a precise argumentation of possible alternatives justifying that they are less suitable than the present one.

Given the exploratory nature of our study examining the relationships among these variables, we employed linear regression analysis using the lm() function in R. This approach was chosen because it allows for a straightforward examination of linear relationships between multiple variables while providing easily interpretable results. While alternative methods such as structural equation modeling (SEM) or generalized additive models (GAM) could be considered, linear regression was more appropriate for our exploratory investigation for several reasons:

1.	Simplicity and Interpretability: Linear regression offers a clear and direct understanding of the relationships between variables without the need for complex model structures, which is ideal for initial explorations.

2.	Model Assumptions and Data Suitability: Our data met most of the necessary assumptions for linear regression, including normality, homoscedasticity, and independence, as demonstrated by our diagnostic tests. In contrast, alternative methods might impose additional assumptions or require transformations that could complicate the analysis without substantial benefits for our study objectives.

3.	Computational Efficiency: Linear regression is computationally less intensive compared to more complex models like SEM, allowing for quicker iterations and analyses during the exploratory phase.

By selecting linear regression, we ensured that our analysis remained focused and interpretable, providing a solid foundation for potential future studies that might employ more advanced techniques as the research questions become more refined.

RESEARCH RESULTS

Why different terms are used: subjective total athletic ability and subjective general athletic ability, subjective general athletic performance and subjective overall motor performance – as this can lead to confusion. It should be unified.

It is incorrect to use the term "total athletic ability" without the word "subjective".

Thank you for your insightful feedback regarding the terminology used in our manuscript. We have addressed the issue of inconsistent terms as follows:

Definitions Clarified:

Subjective general athletic ability: This term refers to a participant's overall self-assessment of their motor competence. L167-168

Subjective general athletic ability, which refers to an individual's self-assessment of their overall physical competence across various types of physical activities and sports,

Subjective total athletic ability: This term represents the sum of subjective athletic abilities across multiple sports, calculated by summing responses to Questions of subjective athletic ability in each sport. L226-227

subjective total motor skill, which is the total subjective athletic abilities score for each discipline,

Correction of Inconsistent Terminology:

The terms "subjective general athletic performance" and "subjective overall motor performance" were typographical errors. We have revised these instances to consistently use either subjective general athletic ability or subjective total athletic ability, as appropriate to the context. Throughout the manuscript, we have ensured that the term "subjective" is included whenever describing data derived from participants' self-assessments. This prevents potential misunderstanding or misrepresentation of the findings. Below are the changes we made.

L28-29: we investigated the determinants of subjective total athletic ability

L33: perceived general athletic ability

L35: perceived total athletic ability

L39: total athletic ability

L220-221: subjective total athletic ability

L224: subjective athletic abilities

L226-227: total subjective athletic abilities

L259-260: subjective general athletic ability

L262-263: subjective total athletic ability

L264-265: subjective total athletic ability
L267-268: subjective total athletic ability.

L270: subjective total athletic ability

L272: subjective total athletic ability

L274: subjective total athletic ability

L275: subjective total athletic ability

L277: subjective total athletic ability

L280: subjective total athletic ability

L282: subjective total athletic ability

L285: subjective total athletic ability

L300-310: subjective total athletic ability.

L309: subjective total athletic ability

L314: subjective total athletic ability

L339: subjective total athletic ability.

L353: subjective total athletic ability

L389: subjective total athletic ability
L390: subjective total athletic ability

L391-392: subjective total athletic ability.

L395: subjective total athletic ability.

L401: subjective total athletic ability.

L404-405: subjective total athletic ability.

L415: subjective total athletic ability

L416: subjective total athletic ability

L429-430: subjective total athletic abilities
L431: subjective total athletic abilities
L432-433: subjective total athletic ability.

L437: subjective total athletic ability
L439: subjective total athletic ability.

L442: subjective total athletic ability

L456: subjective total athletic ability

L437: subjective total athletic ability

L476-477: subjective total athletic ability

L479: subjective total athletic ability
L481: subjective total athletic ability

DISCUSSION PART

Everything must be explained based on the answer to the research question (hypothesis), and in comparison, with other very significant and current studies (2023, 2022) should be clarified much more if the objective has been achieved and why.

We appreciate your suggestion to align the discussion with the research question and hypothesis. We have revised the discussion to explicitly state our hypothesis and provide a clear explanation of how our findings support it. The revised section now includes (L331-L338):

We hypothesized that subjective athletic ability among college students is influenced by personality traits, family environment (e.g., sibling composition), and prior athletic performance. Subjective general athletic ability was highly correlated with subjective athletic ability in short-distance races such as volleyball, basketball, and soccer. Furthermore, various elements such as personality traits, leisure-time activities, and sibling composition were associated with total athletic ability. These findings support our hypothesis that various factors are associated with athletic ability.

As part of our efforts to strengthen the overall quality of the manuscript, we have updated some of the older references with more recent studies, ensuring that the information presented throughout the paper reflects the latest developments in the field.

Please discuss the effect sizes (R2 for regression analysis). Were the observed effects strong/mild/weak? Compare the effect sizes in your data with the effect sizes in previous studies if possible.

Thank you for valuable suggestion. We have reported the coefficient of determination (R²) and adjusted R² in our tables as effect sizes for the multiple regression analyses. Additionally, we have now calculated Cohen's f² values. According to Cohen (1988), f² values of 0.02, 0.15, and 0.35 represent small, medium, and large effect sizes, respectively.

The Cohen's f² values for each model are as follows:

Model 1: f² = 0.222 (medium effect size)

Model 2: f² = 0.394 (large effect size)

Model 3: f² = 0.098 (small to medium effect size)

Model 4: f² = 0.167 (medium effect size)

Model 5: f² = 0.252 (medium effect size)

Model 6: f² = 0.212 (medium effect size)

Model 7: f² = 0.239 (medium effect size)

Model 8: f² = 1.081 (very large effect size)

Notably, Model 2 and Model 8 demonstrated large effect sizes, while the remaining models showed generally medium or higher effect sizes. We have added the effect seize in Table 1 and 2.

We acknowledge the importance of situating our findings within the context of existing research. However, we encountered several challenges in making direct comparisons, which we detail below:

Methodological Differences: Previous studies often employ different statistical models and methods, making it difficult to directly compare their effect sizes with those derived from our regression analyses.

Variability in Study Populations: The demographic and contextual characteristics of study samples can influence the effect sizes reported. For instance, many prior studies examine younger populations or professional athletes, whereas our study focuses on college students.

Differences in Measurement Approaches: Even when addressing similar constructs, prior studies may use distinct measurement tools or scales. This heterogeneity can lead to discrepancies in reported effect sizes that are not due to actual differences in relationships but rather to differences in measurement techniques.

Limited Reporting of Effect Sizes: Many previous studies in this area do not explicitly report R² , Cohen’s f² , or other comparable effect size metrics. This lack of standardized reporting poses a challenge for direct comparisons.

Conceptual Differences: The definition and operationalization of constructs like "athletic ability" or "motor skills" vary significantly across studies, complicating attempts to draw meaningful comparisons.

Given these challenges, we believe that a systematic meta-analysis designed to harmonize definitions, methodologies, and effect size metrics across studies would be invaluable for future research in this domain. We have included this suggestion in the revised manuscript to highlight the need for a more comprehensive and standardized approach to effect size comparisons.

We added the explanation about effect size. (L457-466)

This study identified meaningful effect sizes in the regression analyses, with Cohen’s f² values ranging from small to very large. Notably, Models 2 and 8 showed large or very large effect sizes, underscoring the substantial contribution of the factors examined to variations in subjective athletic ability. However, comparing these effect sizes with prior studies is challenging due to differences in methodologies, study populations, and measurement approaches. Many previous studies also lack detailed reporting of effect sizes. These limitations emphasize the need for a systematic meta-analysis that harmonizes methodologies and standardizes the reporting of effect sizes to facilitate meaningful comparisons. Future research should aim to address these gaps and provide a more unified understanding of the factors influencing athletic ability.

Reviewer #2: Although there are no fatal flaws, there are opportunities to clarify what the study actually achieves and adds to the corpus of scientific literature. The statistical methods could be improved to avoid merely performing numerous associational analyses with minimal structure and organization. The use of clearer language in terms of the constructs being investigated and the conclusions that can be supported by them (throughout the paper) would enhance its rigor. Please see the embedded comments on the manuscript for more details.

We appreciate your positive evaluation of our manuscript. We tried to revise it according to your comments and suggestion. We hope now the manuscript is satisfactorily modified.

P0 Key word: anility ability

We changed the key words to read as (P0)

ability

P3L52-53: from whence are these definitions/quotes derived?

Thank you for your insightful comment. In the revised manuscript, we have clarified that the statement reflects a general understanding rather than a direct quotation from a specific source. To avoid confusion, the term "quotes" has been removed, and the sentence has been rephrased as follows(L51-L54):

Despite frequent references to "He/She is athletic" in daily conversations and sports viewership, the definition is remains vague. This term is commonly understood as an overall evaluation of physical strength and athletic capability as well as the ability to excel across various sports.

P3L62: self-efficacy literature is relevant here

Bandura and those who have built on his work have identified numerous general predictors for the emergence of self-efficacy that would be relevant to the discussion of "motor competence" as a specific manifestation of this

Thank you for your valuable comment. We agree that self-efficacy theory provides a relevant framework for discussing the role of motor competence. In response to your suggestion, we have incorporated references to Bandura's self-efficacy theory and its key predictors (e.g., prior successes, social support, and observational learning) into the discussion. We have revised the sentence as follows (L62-L68):

Confidence resulting from successful exercise experiences (a sense of motor competence) plays a crucial role in improving athletic ability and personality development. This can be understood through the framework of self-efficacy theory proposed by Bandura [9], which identifies prior successes, social support, and observational learning as key predictors for the development of self-efficacy. In this context, motor competence can be viewed as a specific manifestation of self-efficacy that promotes further achievement [10].

P3L64: the transitions between paragraphs could be clearer -- the introduction is lacking in a clear, sequential, and logical flow from one paragraph to the next

Thank you for your valuable feedback. In addition to addressing the comment about paragraph transitions, I also made a few other revisions based on your suggestions. I believe these changes have improved the overall readability and flow of the introduction.

I’ve tried my best to make these adjustments and hope the revised version is clearer now. However, if further revisions are needed, I’d be happy to make additional changes as necessary.

P3L66: it could be clarified whether these terms are believed to be synonymous or representative of different underlying constructs

We appreciate the reviewer's comment regarding the distinction between "motor competence" and "athletic ability." In our study, we have treated these terms as representing distinct constructs:

Motor Competence: Refers to an individual's self-perception of competence or confidence in their ability to perform physical activities. This concept emphasizes subjective evaluations and aligns with definitions in the literature, such as "a measure of perceived physical competence" (Ono & Kaji, 2020, reference number 8).
The above explanation is provided in L61~L62.

Athletic Ability: Refers to the general concept of physical capability, often understood in more objective terms, encompassing elements like strength, speed, coordination, and endurance. This term is widely used in both academic and everyday contexts as a measure of overall athletic performance.

We added above explanation in L70~L72

In this study, athletic ability is defined as the general concept of physical capability, which includes components such as strength, speed, coordination, and endurance.

While motor competence is inherently subjective and tied to self-assessment, athletic ability is often conceptualized and measured more objectively, though subjective interpretations may also exist.

P4L69: source to support claim?

Thank you for your valuable comment. You are correct that the statement regarding the gap in research on college students was not sufficiently supported by sources. As a result, I have removed this claim from the manuscript. Now the part reads as (L73-L75):

While numerous studies have focused on children and adolescents, research on college students remains limited.

P4L79: again, it would be good to clarify whether "motor competence" is being used interchangeably with "athletic ability"

We appreciate the reviewer's comment regarding the distinction between "motor competence" and "athletic ability." In our study, we have treated these terms as representing distinct constructs:

Motor Competence: Refers to an individual's self-perception of competence or confidence in their ability to perform physical activities. This concept emphasizes subjective evaluations and aligns with definitions in the literature, such as "a measure of perceived physical competence" (Ono & Kaji, 2020).

Athletic Ability: Refers to the general concept of physical capability, often understood in more objective terms, encompassing elements like strength, speed, coordination, and endurance. This term is widely used in both academic and everyday contexts as a measure of overall athletic performance.

While motor competence is inherently subjective and tied to self-assessment, athletic ability is often conceptualized and measured more objectively, though subjective interpretations may also exist.

P4L82: ...or that individuals can estimate their physical abilities with relative accuracy

To assert that the subjective evaluation is antecedent to the physical capacity would require establishing temporal precedence -- and, ideally, excluding alternative explanations for the association.

Thank you for your insightful comment. Based on your feedback, I have modified the statement to clarify that subjective athletic ability may be influenced by sports experience, and that individuals can estimate their physical abilities with reasonable accuracy. Now the part reads as (L92-L94):

This indicates that differences in sports experience may significantly influence subjective athletic ability, or that individuals can estimate their physical abilities with relative accuracy.

P4L90: "...is the belief that talent can be developed with consistent efforts..."

(I think this would read better, but it is subjective.)

We changed the sentence to read as (L102-L103)

and a growth mindset is the belief that talent can be developed consistent efforts to improve one’s ability

P4L91: I do not agree with this wording. One cannot have "competencies in personality traits". Perhaps it would be better to state what I believe is intended more directly. Namely, the author is asserting that higher levels of psychological factors such as grit, resilience, and growth mindset may be associated with persistence in sport which in turn is likely to enhance sport performance and develop athletic ability."

(Of note, this is a hypothesis for mediation which would be interesting to test.)

Thank you for pointing out the need for more precise wording. We agree that the term "competencies in personality traits" is not appropriate. In response, we have revised the sentence to reflect the intended meaning more clearly by focusing on psychological factors and their potential influence on persistence in sports and athletic performance. The revised sentence reads (L103-L106):

Higher levels of psychological factors such as grit, resilience, and a growth mindset may be associated with greater persistence in sports, which in turn is likely to enhance athletic performance and develop athletic ability and motor competence.

P5L106: I assume this is SD given descriptive statistics are being reported, but it would be ideal to specify as +/- could also refer to SE.

Thank you for your suggestion. Yes, these values are standard deviations (SD). We have modified the notation in our manuscript to clearly specify SD rather than using +/-. The part now reads as (L125).

(mean age: 19.31 years, SD: 0.95; female: 221, male: 185).

P5L110: Can the authors clarify the combination of consent being "waived by the ethics committee" with the obtainment of informed consent from participants? I am not sure how informed consent would be "waived" by an ethics committee.

Thank you for pointing out the potential confusion in our wording. Upon review, we have revised the text to clarify this point. Specifically, informed consent was obtained from all participants via Google Forms. For minors involved in the study, the ethics committee waived the requirement to obtain parental or guardian consent. The revised text now reads: (L127-L130)

In this study, informed consent was obtained from all participants via Google Forms (Alphabet Inc., U.S.A.). For minors involved in the study, informed consent was obtained directly from the participants, while the requirement for parental or guardian consent was waived by the ethics committee.

P7L164: It may be possible to provide the scale construction details in a supplementary file or such to help increase the readability/flow of the Method.

Thank you for your suggestion regarding the scale construction details. We would like to address this point as follows:

The questionnaire used in this study was constructed via Google Forms and organized into multiple sections, resulting in a detailed yet complex structure. Presenting the full construction details may overwhelm readers due to the sheer volume and intricacy of the information.

Furthermore, as the original questionnaire was created in Japanese, including it directly may pose challenges for non-Japanese-speaking readers, and we are concerned it could detract from the overall readability of the manuscript.

However, if the reviewer feels that providing this information as supplementary material would enhance the manuscript's clarity and utility, we are more than willing to include it upon request.

P8L169: ...and what were the findings regarding its validity?]

Thank you for your comment. Nishikawa et al. reported that the Japanese version of the Grit-S scale demonstrates a factor structure and reliability that closely resemble those of the original Grit-S scale. Additionally, the Japanese version showed evidence of basic validity, indicating its suitability for assessing grit in a Japanese-speaking population. These findings support the use of the translated scale in our study as a valid measure of the grit construct. Now the part reads as (L185-L187):

Nishikawa et al. assessed the validity of the questionnaire translated into Japanese, reporting that it retained a reliability, and basic validity as the original short Grit scale [28].

P8L175: Again, details regarding the validity from these studies would be ideal. It needn't be long, but a statement such as: "Tokuyoshi and Moriya [29] found that the Japanese version of the scale showed [low/acceptable/high] [construct/concurrent/predictive/etc.] validity" would be better than simply saying they assessed it.

Thank you for your suggestion. We have added specific details regarding the validity of the scale as reported in Tokuyoshi and Moriya's study. They confirmed that the Japanese version of the scale demonstrated acceptable levels of construct validity, ensuring its applicability within the context of this study. Now the part reads as (L192-L194):

Tokuyoshi and Moriya found that the Japanese version of the scale demonstrated acceptable construct validity, supporting its use for assessing relevant constructs in Japanese populations [30].

P8L182: Without the need to be exhaustive, it would be helpful to give a couple of examples of the items that were chosen to represent "growth mindset" from the scale.

Thank you for your suggestion. To address this, we have included examples of the items selected from the Intelligence Scale to represent a "growth mindset." These examples help clarify how the construct was assessed in this study. Now the part reads as (L200-L203):

Ten items from the Intelligence Scale were used in this study [31]. Examples of the items include "Intelligence is an innate ability determined at birth" and "I believe that I can become smarter through effort."

P9L202: The excessive use of correlation and linear regressions is somewhat questionable. If the issue was one of selection in a exploratory sense from a large number of predictors, why not use a model meant to perform selection (e.g., lasso among various options comes to mind). There are also more "traditional" approaches in linear regression such as forward or backward stepwise modeling, etc.

Thank you for your thoughtful suggestion regarding alternative statistical approaches. In this study, our primary aim was to explore the influence of variables on our outcome measures within the same category, based on reference data in our dataset. Therefore, we intentionally chose not to employ variable selection methods such as LASSO regression with penalty terms or model selection based on AIC/BIC criteria. Instead, we used traditional linear regression to directly examine the relationships between our variables of interest. Additionally, we tested the key assumptions for linear regression across all models, including independence (The Durbin-Watson Statistic) and homoscedasticity (The Breusch-Pagan test), which confirmed the appropriateness of our analytical approach.

P9L207: I do not understand why this is made into a new paragraph when the previous sentence and this one are clearly, directly connected. It would be more reasonable to do the paragraph break before the last sentence of the previous paragraph.

We agree that the sentences are directly connected and should not have been split into separate paragraphs. To improve readability and maintain the logical flow of the text, we have combined the sentences into the same paragraph. The revised text now reads: (L231-L233)

Japan uses a unique annual age-grouping policy for sports and education, effective between April 1 to March 31 of the following year. Consequently, for the birth month, the numbers were adjusted such that April births began at 1 and March births ended at 12.

P9L211: Do the authors mean that it was the reference category for the leisure time variable? A criterion variable is an outcome or "dependent" variable in linear regression.

Thank you for your comment. Regarding the reference categories, we have provided detailed information about all categorical variables and their respective reference categories in the Methods section (lines L237-L239). This includes the specific reference category for the leisure time variable.

P9L212: Again, this phrasing does not seem correct to me. I think "reference category" is intended.

We changed the sentence to read as: (L240-L241)

and the reference category was set as an only child.

P10L216: category

We changed the sentence to read as: (L243-L245)

A dummy variable was used for competition performance and the reference category was the qualification level required for the prefectural tournament.

P10L224: reference category

We changed the sentence to read as: (L251-L252)

and the reference category was "never been told."

P10L226: reference category

We changed the sentence to read as: (L253-L254)

with the reference category being "very slow compared with the average person."

P10L229: Why are the authors trying to assign specific month values to these vague responses, especially when they end up doing a variable split to categorize participant responses into what seems to be three groups? I think it would be clearer to simply state: "Three groups were constructed for those who reported the age at which they first walked, 1) <9 months, 2) 9-17 months, or 3) >17 months. Those who reported "on the early side," about average," or "on the late side" were put into these three brackets, respectively." As a note, using 9 months is very early for creating the "middle" bracket. 8-18 months is considered developmentally normal for walking. Can the authors provide a source to justify this categorization approach?

Thank you for your valuable comments regarding the walking categorization. After careful consideration, we have decided to remove the walking age analysis from our study for the following reasons:

1. **Data Reliability**

The self-reported walking initiation data were primarily based on vague responses such as "on the early side" or "on the late side." These responses do not provide precise month values, making it difficult to ensure the reliability of the data. As a result, we were unable to obtain consistent, accurate data across participants.

2. **Categorization Challenges**
   The developmental normal range for walking initiation, which spans 8-18 months, overlaps with our attempted categorization. This makes it difficult to clearly define what constitutes "early" or "late" walking age in a meaningful way.

Given these limitations, we believe this variable is unsuitable for rigorous statistical analysis. Instead, we have revised our analysis to focus on other, more reliably measured outcomes.

P11L243: specifying the traits would be useful here for clarity (i.e., higher scores for grit, resilience, and growth mindset)

Thank you for your suggestion. We changed the sentence to read as: (L263-L265)

Model 1 demonstrated that higher scores for grit, resilience and growth mindset were associated with greater subjective total athletic ability

P12L263: It would be worth noting that these are subjective evaluations and recollections; as worded, it sounds like it was measured objectively

We changed the sentence to read as: (L285)

Subjective total athletic ability

P15 Table2: For the variables entered into the model as categorical, it would be nice to see a contrast specified to test the multiple degrees of freedom categorical effect to answer whether the "variable" as a whole was a significant predictor.

Thank you for your suggestion regarding the testing of categorical variables' overall effects. We have conducted Type III tests to evaluate whether each categorical variable as a whole was a significant predictor in our model. The results of these analyses have been included in the supplementary materials (Supplementary Table X).

This additional analysis provides a comprehensive assessment of each categorical variable's overall significance in the model, addressing the multiple degrees of freedom categorical effects you mentioned.

P17 Table2: With so many models performed it is questionable that there was no sequential or selection modeling approach taken. It is also notable that the sample being used differs dramatically across models with as few as 150 participants to the full 406 who identified their gender (as male or female) and consented.

Thank you for these important points. As mentioned in our previous response, this study took an exploratory data analysis approach to examine the relationships between variables within specific categories, rather than employing variable selection methods. The variation in sample sizes across models reflects our use of listwise deletion for handling missing data, which we believe was appropriate for this exploratory investigation.

P18L294: The authors continually refer to the idea of identifying "motor skills" or "athletic ability," but I would argue that little has been done to advance understanding of this in the absence of any concrete, objective measures of such ability. If the desire is to identify what is meant in the general population when they say someone possesses "athletic ability," then the task would be to develop items through some focus groups and literature review and test them in large sample to engage in scale construction via factor analytic or other methods. If the goal is to identify correlates of "athletic ability" in developmental processes, I would argue it is essential to obtain objective measures of "athletic ability" to assert that you have identified true correlates of that ability. Further, such an approach would be enhanced greatly by identifying predictors -- not just correlates -- by using a time series approach to support the notion of a developmental trajectory or pathway occurring in the hypothesized order. This study has not taken either approach which causes it to falter in its attempt to advance the understanding of "motor skill" or "athletic ability" in the way it purports is worthwhile.

Thank you for your insightful comments. We understand your concerns regarding the limitations of using subjective measures of "athletic ability" and the need for objective assessments in advancing our understanding of this concept.

As you correctly pointed out, this study was exploratory in nature, and our primary goal was to examine the relationship between subjective athletic ability and various factors such as personality traits, sibling composition, and athletic performance. However, we acknowledge the importance of using concrete, objective measures to accurately assess "athletic ability." While the current study relied on self-reported data, we have explicitly noted the limitations of such an approach, particularly in relation to the challenges of defining and measuring subjective athletic ability with precision.

To address your point about the need for objective measures, we have included a discussion of the study's limitations in the revised manuscript. Specifically, we have highlighted that future research should incorporate objective evaluation indices, as well as longitudinal designs, to provide more robust data on the developmental trajectories of athletic ability and motor skills. These recommendations are aimed at bridging the gap between subjective perceptions and objective performance metrics, as well as identifying predictors and correlates of athletic ability.

We have also revised the limitations section to emphasize that this study's reliance on cross-sectional data restricts our ability to infer causal relationships between the identified factors and athletic ability. We are aware of the need for a more detailed, longitudinal approach to capture the developmental aspects of motor skills and athletic performance.

In summary, while this study contributes to the current understanding of subjective athletic ability, we recognize the need for more rigorous methods in future research to confirm and expand upon these findings. We appreciate your feedback and hope the revised manuscript addresses your concerns effectively. Now the part reads as :

Purpose (L107-L117)
Therefore, this study aimed to explore the relationship between subjective athletic ability and selected factors such as personality traits, sibling composition, and athletic performance among college students. Given the exploratory nature of this study, we examined potential influencing factors on subjective athletic ability from various perspectives, acknowledging the limitations inherent in self-reported measures and the challenges of defining subjective athletic ability with precision.

Limitation (L467-L484)
This study has some limitations. There may be some bias in the parents' income and sample because this study was conducted only with students at a particular college. Moreover, parents' athletic abilities affect their children's athletic abilities; however, this influence cannot be completely eliminated. In addition, this study was conducted with participants who subjectively evaluated their athletic abilities. The study's reliance on self-reported data and its exploratory design limit its ability to provide concrete, objective measures or developmental trajectories of athletic ability and motor skills. In the future, more detailed studies will be possible by combining objective evaluation indices. Furthermore, as this study used a cross-sectional design to obtain data, we could not interpret the causal relationship between the factors associated with total athletic ability found in this study. A longitudinal study is needed to determine changes in total athletic ability to identify factors that may enhance motor skills. This study examined the factors associated with motor skills by employing various types of linear models; however, it did not ascertain the extent to which each factor contributed to total athletic ability. Therefore, additional studies are required to confirm these findings. Future research should aim to address these gaps by utilizing validated measures, conducting longitudinal studies, and incorporating objective performance metrics.

P19L334: I can understand the assertion that there is a lesser degree of skill acquisition in short-distance running, but I think some would find it objectionable to assert that it does not involve the acquisition of skill above and beyond simple physical ability.

Thank you for your comment. I understand your concern about asserting that short-distance racing involves only physical ability and not skill. To address this, I have revised the statement to reflect that while short-distance racing tends to be more about physical ability compared to other sports, it does not exclude the role of skill acquisition. The revised sentence now includes the phrase "tends to" to make the assertion less absolute. Now the part reads as (L367-L369):

However, compared with other sports, short-distance racing tends to reflect an individual’s physical ability more than skill and experience. Further studies are required to determine the effects of short-distance racing ability on other sports.

P21L376: You collected data regarding age of walking onset and birth order. It would be interesting to note whether your study replicated this finding that has been reported previously.

Thank you for your suggestion regarding the age of walking onset and birth order. Upon reviewing the data and considering other revisions to the manuscript, I have decided to remove the mention of walking onset age from the study.

P22L382: That is quite a leap of generalization. Perhaps the phrase "may be" would be worth adding.

We added the phrase "may be" in the sentence. Now the part reads as (L416-L417):

Considering that sibling composition is associated with subjective total athletic ability, family involvement may be a significant factor in the development of motor skills.

P22L384: "The effect of parental athleticism on child athleticism occurs via both genetic and environmental processes."

Thank you for your valuable suggestion and we revised the sentence. Now the part reads as (L419-L420):

The effect of parental athleticism on child athleticism occurs via both genetic and environmental processes.

P23L410: Although it is possible that age of walking initiation does, in fact, "improve physical ability of college students," this cannot be said from this study. Studies the authors cite demonstrate the association between this variable and other objectively measured physical outcomes (e.g,. grip strength), but this study only associated it with subjective evaluations and self-reported abilities. Second, it is also possible that both the age of walking initiation and these other variables (whether they be the objectively measured constructs in cited research or the subjectively appraised ones in this study) are both caused by underlying genetic abilities. As such, the correlation between these could be spurious -- at minimum, it is certainly reasonable to suggest that age of walking onset may not be causal in the development of physical abilities in later life as the phrasing used here implies.

Thank you for your suggestion regarding the age of walking onset and birth order. Upon reviewing the data and considering other revisions to the manuscript, I have decided to remove the mention of walking onset age from the study.

P23L413: Researchers? Perhaps observers?

We changed the words "other researchers" to "others". Now the part reads as (L439-L440):

The results obtained in this study indicate the importance of evaluations by others.

P23L415: There are many possible pathways for this. It could be that others and selves are both simply observing the same underlying ability objectively and not directly influencing one another. It is also possible that others' views of one's ability shape one's own perception of his/her ability (e.g., Cooley's looking glass self theory of identity formation). Lastly, because -- importantly -- the authors did not actually measure others' perceptions, rather they measured the individual's report of what they perceive and recall of others' perceptions, it is possible that the view one holds of one's own abilities biases and distorts perceptions and recollections of feedback they've received from others (e.g., confirmation bias among other processes that have been well-established in psychological literature regarding the malleability of memory and perceptual processes based on present personal states and expectations).

Thank you for your insightful comments. We appreciate your suggestions regarding the complexity of the relationship between others' evaluations and subjective total athletic ability. We recognize that the association may involve multiple factors, and we have revised the manuscript to reflect a more nuanced view. Specifically, we have addressed the possibility that the association between evaluations by others and subjective athletic ability could be influenced by cognitive processes such as confirmation bias, as well as the potential role of self-perception influenced by others' evaluations, as described by Cooley's looking-glass self theory. We have also acknowledged the limitations of our study, particularly with regard to our reliance on self-reported data about others' perceptions. Now the part reads as (L452-L451):

However, it is important to acknowledge that there are various possible explanations for this association. It could be that both individuals and others are simply observing the same underlying ability objectively, with no direct influence between the two. Alternatively, others' views of one's ability may shape one's self-perception, as suggested by Cooley's looking-glass self theory of identity formation [49]. Since our study did not directly measure others' perceptions but instead relied on individuals' reports of what they perceive and recall about others' evaluations, it is also possible that participants' views of their own abilities influenced their recollections and perceptions of feedback they received, a phenomenon consistent with well-established psychological theories such as confirmation bias [50].

P23L420: In general, the discussion needs to be phrased much more carefully given the notable limits of what was actually measured in the current study.

We appreciate the reviewer’s observation regarding the need for more cautious phrasing in light of the study’s limitations. In response, we have carefully reviewed and revised the discussion section to address this concern. Specifically:

Clarifying Study Limitations: As noted, we have acknowledged the notable limitations of our study in the revised discussion. Claims based on less reliable data or indirect measurements have been either removed or framed more cautiously to ensure they align with the study’s scope and findings.

Strengthening Terminological Precision: To prevent potential misinterpretation, we have clarified the definitions of key terms throughout the manuscript. This ensures that the use of terminology such as "subjective athletic ability," "motor competence," and related constructs accurately reflects their intended meaning within the context of this study.

P24L428: Yes, but the phrasing that is used does not capture this sufficiently in its present form. Further, the language used to describe the variables that were actually measured needs to be crafted more carefully, too.

We appreciate the reviewer’s insightful feedback regarding the phrasing and clarity of our limitations section. In response, we have revised the paragraph to better capture the study’s constraints and ensure that the description of the variables measured is more precise. Specifically:

We have refined the language to clearly delineate the limitations of self-reported data and the exploratory nature of the study.

The revised text more accurately addresses the implications of using subjective evaluations and acknowledges the methodological constraints of our study design. Now the limitation part reads as (L467-L484):

This study has some limitations. There may be some bias in the parents' income and sample because this study was conducted only with students at a particular college. Moreover, parents' athletic abilities affect their children's athletic abilities; however, this influence cannot be completely eliminated. In addition, this study was conducted with participants who subjectively evaluated their athletic abilities. The study's reliance on self-reported data and its exploratory design limit its ability to provide concrete, objective measures or developmental trajectories of athletic ability and motor skills. In the future, more detailed studies will be possible by combining objective evaluation indices. Furthermore, as this study used a cross-sectional design to obtain data, we could not interpret the causal relationship between the factors associated with subjective total athletic ability found in this study. A longitudinal study is needed to determine changes in total athletic ability to identify factors that may enhance motor skills. This study examined the factors associated with subjective total athletic ability by employing various types of linear models; however, it did not ascertain the extent to which each factor contributed to subjective total athletic ability. Therefore, additional studies are required to confirm these findings. Future research should aim to address these gaps by utilizing validated measures, conducting longitudinal studies, and incorporating objective performance metrics.

Additional Revisions Made to the Manuscript

In addition to addressing the reviewers' comments, we have made a few additional revisions to enhance the clarity and accuracy of the manuscript. These changes are as follows:

Figure 3: Corrected the model numbering to align with the text and other figures.

Figure 2: Revised Model 5 to ensure consistency with the results presented in the analysis.

Figure Titles: Updated the titles of Figures 2 and 3 to provide clearer descriptions of the content.

These modifications were made to improve the quality and presentation of the manuscript. They do not alter the study's findings or conclusions. We hope these revisions contribute to a more precise and comprehensible representation of our work.
